# Supplementary material for: Digital health in fragile states in the Middle East and North Africa (MENA) region: A scoping review of the literature
Source: PLoS One. 2023 Apr 28;18(4):e0285226. doi: 10.1371/journal.pone.0285226 (PMC10146476; doi:10.1371/journal.pone.0285226)
Supplement: S1 Appendix — (DOCX) [file pone.0285226.s002.docx]

S1 Appendix: Search strategies

Medline Search Strategy

Database: Ovid MEDLINE(R) and Epub Ahead of Print, In-Process, In-Data-Review & Other Non-Indexed Citations and Daily <1946 to October 14, 2022>

Search Strategy:

--------------------------------------------------------------------------------

1 middle east/ or Africa, Northern/ or Afghanistan/ or Algeria/ or Djibouti/ or egypt/ or libya/ or morocco/ or tunisia/ or iran/ or iraq/ or jordan/ or lebanon/ or Somalia/ or Pakistan/ or Sudan/ or syria/ or yemen/ (130441)

2 (Afghanistan or Algeria or Djibouti or Egypt or Libya or morocco or Tunisia or Iran or Iraq or Jordan or Lebanon or Palestine or Gaza or Pakistan or Somalia or Somaliland or Sudan or Syria or (syrian adj arab* adj republic) or (west adj bank) or Yemen).ti,ab. (143400)

3 ((middle adj east) or (north* adj Africa)).ti,ab. (18216)

4 mena.ti,ab. (1089)

5 ((fragile or weak or fragility or conflict or conflicts or conflict-affected or war or wars or war-affected or post-conflict? or post-war or post-wars) adj (setting? or context or contexts or state or states or country or countries)).ti,ab. (1094)

6 Mediterranean Region/ (3945)

7 (East* adj Mediterranean).ti,ab. (3422)

8 exp Telemedicine/ or medical records systems, computerized/ or electronic health records/ or health information exchange/ or health smart cards/ or vital statistics/ or sentinel surveillance/ or information science/ or internet/ or information services/ or information technology/ or "diffusion of innovation"/ or Cloud Computing/ or Medical Informatics/ (208546)

9 ((Digital or Digitized) adj2 health).ti,ab. (4548)

10 (cloud-based adj2 system?).ti,ab. (145)

11 ICT.ti,ab. (6911)

12 (information adj2 (system or systems or technology)).ti,ab. (57355)

13 (civil registration and vital statistics).ti,ab. (152)

14 ((electronic or mobile) adj health).ti,ab. (30923)

15 (m-health or mhealth or e-health or ehealth).ti,ab. (11468)

16 ((health or wellness or mobile) adj (app or apps or application or applications)).ti,ab. (9997)

17 "health 2.0".ti,ab. (127)

18 "medicine 2.0".ti,ab. (117)

19 ((Privacy or security or protection or confidentiality) adj2 (data or information or internet)).ti,ab. (8342)

20 (electronic adj2 (record or records)).ti,ab. (52715)

21 (Telemedicine or telecare or telehealth).ti,ab. (25107)

22 Interoperability.ti,ab. (4503)

23 Health Information Interoperability/ (225)

24 "big data".ti,ab. (10427)

25 (health adj analytics).ti,ab. (321)

26 or/1-7 (213747)

27 or/8-25 (330603)

28 26 and 27 (3135)

29 limit 28 to ez="20200831-20220831" (763)

30 limit 28 to yr="2000 -Current" (1600)

***************************

PubMed Search Strategy

Update

| Search number | Query | Sort By | Filters | Search Details | Results | Time |
| --- | --- | --- | --- | --- | --- | --- |
| 20 | #17 AND #19 | |  | ("Telemedicine"[MeSH Terms] OR "Telemedicine"[Title/Abstract] OR "telecare"[All Fields] OR ("telehealth s"[All Fields] OR "Telemedicine"[MeSH Terms] OR "Telemedicine"[All Fields] OR "telehealth"[All Fields]) OR "m-health"[All Fields] OR ("mhealth s"[All Fields] OR "Telemedicine"[MeSH Terms] OR "Telemedicine"[All Fields] OR "mhealth"[All Fields]) OR "e-health"[All Fields] OR ("Telemedicine"[MeSH Terms] OR "Telemedicine"[All Fields] OR "ehealth"[All Fields]) OR ("interoperability"[All Fields] OR "interoperable"[All Fields] OR "interoperate"[All Fields] OR "interoperates"[All Fields] OR "interoperating"[All Fields] OR "interoperation"[All Fields]) OR ("digital health"[Title/Abstract] OR "digitized health"[Title/Abstract]) OR ("information system"[Title/Abstract] OR "information systems"[Title/Abstract] OR "information technology"[Title/Abstract] OR "information technologies"[Title/Abstract] OR "communication technology"[Title/Abstract]) OR ("cloud based system"[Title/Abstract] OR "cloud based systems"[Title/Abstract]) OR "ICT"[Title/Abstract] OR "civil registration and vital statistics"[Title/Abstract] OR ("electronic health"[Title/Abstract] OR "mobile health"[Title/Abstract]) OR ("health app"[Title/Abstract] OR "health apps"[Title/Abstract] OR "health application"[Title/Abstract] OR "mobile app"[Title/Abstract] OR "mobile apps"[Title/Abstract] OR "mobile application"[Title/Abstract] OR "mobile applications"[Title/Abstract]) OR ("health 2 0"[Title/Abstract] OR "medicine 2 0"[Title/Abstract]) OR ("data privacy"[Title/Abstract] OR "data security"[Title/Abstract] OR "data protection"[Title/Abstract] OR "data confidentiality"[Title/Abstract] OR "information privacy"[Title/Abstract] OR "information security"[Title/Abstract] OR "information protection"[Title/Abstract] OR "information confidentiality"[Title/Abstract]) OR ("electronic health record"[Title/Abstract] OR "electronic health records"[Title/Abstract] OR "electronic medical record"[Title/Abstract] OR "electronic medical records"[Title/Abstract]) OR ("health analytics"[Title/Abstract] OR "data analytics"[Title/Abstract] OR "big data"[Title/Abstract])) AND ("fragile state"[Title/Abstract] OR "fragile states"[Title/Abstract] OR ("fragile"[All Fields] AND "country"[Title/Abstract]) OR "fragile countries"[Title/Abstract] OR "fragile setting"[Title/Abstract] OR "fragile settings"[Title/Abstract] OR "fragile context"[Title/Abstract] OR "fragile contexts"[Title/Abstract] OR "weak state"[Title/Abstract] OR "conflict states"[Title/Abstract] OR "conflict state"[Title/Abstract] OR "conflict area"[Title/Abstract] OR "conflict areas"[Title/Abstract] OR "conflict country"[Title/Abstract] OR "conflict countries"[Title/Abstract] OR "conflict setting"[Title/Abstract] OR "conflict settings"[Title/Abstract] OR "conflict context"[Title/Abstract] OR "conflict contexts"[Title/Abstract] OR "conflict-affected"[Title/Abstract] OR "war setting"[Title/Abstract] OR "war settings"[Title/Abstract] OR "war context"[Title/Abstract] OR "war contexts"[Title/Abstract] OR (("armed conflicts"[MeSH Terms] OR ("armed"[All Fields] AND "conflicts"[All Fields]) OR "armed conflicts"[All Fields] OR "war"[All Fields]) AND "countries"[Title/Abstract]) OR "war-affected"[All Fields] OR "post-conflict"[All Fields] OR "post-war"[All Fields] OR "post-wars"[Title/Abstract] OR ("Middle East"[All Fields] OR "Middle East"[Title/Abstract] OR "eastern mediterranean"[Title/Abstract] OR "Afghanistan"[Title/Abstract] OR "Algeria"[Title/Abstract] OR "Djibouti"[Title/Abstract] OR "Egypt"[Title/Abstract] OR "Libya"[Title/Abstract] OR "morocco"[Title/Abstract] OR "Tunisia"[Title/Abstract] OR "Iran"[Title/Abstract] OR "Iraq"[Title/Abstract] OR "Jordan"[Title/Abstract] OR "Lebanon"[Title/Abstract] OR "Palestine"[Title/Abstract] OR "Gaza"[Title/Abstract] OR "Pakistan"[Title/Abstract] OR "Somalia"[Title/Abstract] OR "Somaliland"[Title/Abstract] OR "Sudan"[Title/Abstract] OR "Syria"[Title/Abstract] OR "syrian arab republic"[Title/Abstract] OR "west bank"[Title/Abstract] OR "Yemen"[Title/Abstract])) AND 2020/08/01:2022/08/31[Date - Create] | 794 | 5:32:36 |
| 19 | 2020/08:2022/08 [crdt] | | | 2020/08/01:2022/08/31[Date - Create] | 3,269,619 | 5:32:10 |
| 18 | #15 AND #16 | | from 2020 - 2022 | (("Telemedicine"[MeSH Terms] OR "Telemedicine"[Title/Abstract] OR "telecare"[All Fields] OR ("telehealth s"[All Fields] OR "Telemedicine"[MeSH Terms] OR "Telemedicine"[All Fields] OR "telehealth"[All Fields]) OR "m-health"[All Fields] OR ("mhealth s"[All Fields] OR "Telemedicine"[MeSH Terms] OR "Telemedicine"[All Fields] OR "mhealth"[All Fields]) OR "e-health"[All Fields] OR ("Telemedicine"[MeSH Terms] OR "Telemedicine"[All Fields] OR "ehealth"[All Fields]) OR ("interoperability"[All Fields] OR "interoperable"[All Fields] OR "interoperate"[All Fields] OR "interoperates"[All Fields] OR "interoperating"[All Fields] OR "interoperation"[All Fields]) OR ("digital health"[Title/Abstract] OR "digitized health"[Title/Abstract]) OR ("information system"[Title/Abstract] OR "information systems"[Title/Abstract] OR "information technology"[Title/Abstract] OR "information technologies"[Title/Abstract] OR "communication technology"[Title/Abstract]) OR ("cloud based system"[Title/Abstract] OR "cloud based systems"[Title/Abstract]) OR "ICT"[Title/Abstract] OR "civil registration and vital statistics"[Title/Abstract] OR ("electronic health"[Title/Abstract] OR "mobile health"[Title/Abstract]) OR ("health app"[Title/Abstract] OR "health apps"[Title/Abstract] OR "health application"[Title/Abstract] OR "mobile app"[Title/Abstract] OR "mobile apps"[Title/Abstract] OR "mobile application"[Title/Abstract] OR "mobile applications"[Title/Abstract]) OR ("health 2 0"[Title/Abstract] OR "medicine 2 0"[Title/Abstract]) OR ("data privacy"[Title/Abstract] OR "data security"[Title/Abstract] OR "data protection"[Title/Abstract] OR "data confidentiality"[Title/Abstract] OR "information privacy"[Title/Abstract] OR "information security"[Title/Abstract] OR "information protection"[Title/Abstract] OR "information confidentiality"[Title/Abstract]) OR ("electronic health record"[Title/Abstract] OR "electronic health records"[Title/Abstract] OR "electronic medical record"[Title/Abstract] OR "electronic medical records"[Title/Abstract]) OR ("health analytics"[Title/Abstract] OR "data analytics"[Title/Abstract] OR "big data"[Title/Abstract])) AND ("fragile state"[Title/Abstract] OR "fragile states"[Title/Abstract] OR ("fragile"[All Fields] AND "country"[Title/Abstract]) OR "fragile countries"[Title/Abstract] OR "fragile setting"[Title/Abstract] OR "fragile settings"[Title/Abstract] OR "fragile context"[Title/Abstract] OR "fragile contexts"[Title/Abstract] OR "weak state"[Title/Abstract] OR "conflict states"[Title/Abstract] OR "conflict state"[Title/Abstract] OR "conflict area"[Title/Abstract] OR "conflict areas"[Title/Abstract] OR "conflict country"[Title/Abstract] OR "conflict countries"[Title/Abstract] OR "conflict setting"[Title/Abstract] OR "conflict settings"[Title/Abstract] OR "conflict context"[Title/Abstract] OR "conflict contexts"[Title/Abstract] OR "conflict-affected"[Title/Abstract] OR "war setting"[Title/Abstract] OR "war settings"[Title/Abstract] OR "war context"[Title/Abstract] OR "war contexts"[Title/Abstract] OR (("armed conflicts"[MeSH Terms] OR ("armed"[All Fields] AND "conflicts"[All Fields]) OR "armed conflicts"[All Fields] OR "war"[All Fields]) AND "countries"[Title/Abstract]) OR "war-affected"[All Fields] OR "post-conflict"[All Fields] OR "post-war"[All Fields] OR "post-wars"[Title/Abstract] OR ("Middle East"[All Fields] OR "Middle East"[Title/Abstract] OR "eastern mediterranean"[Title/Abstract] OR "Afghanistan"[Title/Abstract] OR "Algeria"[Title/Abstract] OR "Djibouti"[Title/Abstract] OR "Egypt"[Title/Abstract] OR "Libya"[Title/Abstract] OR "morocco"[Title/Abstract] OR "Tunisia"[Title/Abstract] OR "Iran"[Title/Abstract] OR "Iraq"[Title/Abstract] OR "Jordan"[Title/Abstract] OR "Lebanon"[Title/Abstract] OR "Palestine"[Title/Abstract] OR "Gaza"[Title/Abstract] OR "Pakistan"[Title/Abstract] OR "Somalia"[Title/Abstract] OR "Somaliland"[Title/Abstract] OR "Sudan"[Title/Abstract] OR "Syria"[Title/Abstract] OR "syrian arab republic"[Title/Abstract] OR "west bank"[Title/Abstract] OR "Yemen"[Title/Abstract]))) AND (2020:2022[pdat]) | 1,020 | 5:30:05 |
| 17 | #15 AND #16 | |  | ("Telemedicine"[MeSH Terms] OR "Telemedicine"[Title/Abstract] OR "telecare"[All Fields] OR ("telehealth s"[All Fields] OR "Telemedicine"[MeSH Terms] OR "Telemedicine"[All Fields] OR "telehealth"[All Fields]) OR "m-health"[All Fields] OR ("mhealth s"[All Fields] OR "Telemedicine"[MeSH Terms] OR "Telemedicine"[All Fields] OR "mhealth"[All Fields]) OR "e-health"[All Fields] OR ("Telemedicine"[MeSH Terms] OR "Telemedicine"[All Fields] OR "ehealth"[All Fields]) OR ("interoperability"[All Fields] OR "interoperable"[All Fields] OR "interoperate"[All Fields] OR "interoperates"[All Fields] OR "interoperating"[All Fields] OR "interoperation"[All Fields]) OR ("digital health"[Title/Abstract] OR "digitized health"[Title/Abstract]) OR ("information system"[Title/Abstract] OR "information systems"[Title/Abstract] OR "information technology"[Title/Abstract] OR "information technologies"[Title/Abstract] OR "communication technology"[Title/Abstract]) OR ("cloud based system"[Title/Abstract] OR "cloud based systems"[Title/Abstract]) OR "ICT"[Title/Abstract] OR "civil registration and vital statistics"[Title/Abstract] OR ("electronic health"[Title/Abstract] OR "mobile health"[Title/Abstract]) OR ("health app"[Title/Abstract] OR "health apps"[Title/Abstract] OR "health application"[Title/Abstract] OR "mobile app"[Title/Abstract] OR "mobile apps"[Title/Abstract] OR "mobile application"[Title/Abstract] OR "mobile applications"[Title/Abstract]) OR ("health 2 0"[Title/Abstract] OR "medicine 2 0"[Title/Abstract]) OR ("data privacy"[Title/Abstract] OR "data security"[Title/Abstract] OR "data protection"[Title/Abstract] OR "data confidentiality"[Title/Abstract] OR "information privacy"[Title/Abstract] OR "information security"[Title/Abstract] OR "information protection"[Title/Abstract] OR "information confidentiality"[Title/Abstract]) OR ("electronic health record"[Title/Abstract] OR "electronic health records"[Title/Abstract] OR "electronic medical record"[Title/Abstract] OR "electronic medical records"[Title/Abstract]) OR ("health analytics"[Title/Abstract] OR "data analytics"[Title/Abstract] OR "big data"[Title/Abstract])) AND ("fragile state"[Title/Abstract] OR "fragile states"[Title/Abstract] OR ("fragile"[All Fields] AND "country"[Title/Abstract]) OR "fragile countries"[Title/Abstract] OR "fragile setting"[Title/Abstract] OR "fragile settings"[Title/Abstract] OR "fragile context"[Title/Abstract] OR "fragile contexts"[Title/Abstract] OR "weak state"[Title/Abstract] OR "conflict states"[Title/Abstract] OR "conflict state"[Title/Abstract] OR "conflict area"[Title/Abstract] OR "conflict areas"[Title/Abstract] OR "conflict country"[Title/Abstract] OR "conflict countries"[Title/Abstract] OR "conflict setting"[Title/Abstract] OR "conflict settings"[Title/Abstract] OR "conflict context"[Title/Abstract] OR "conflict contexts"[Title/Abstract] OR "conflict-affected"[Title/Abstract] OR "war setting"[Title/Abstract] OR "war settings"[Title/Abstract] OR "war context"[Title/Abstract] OR "war contexts"[Title/Abstract] OR (("armed conflicts"[MeSH Terms] OR ("armed"[All Fields] AND "conflicts"[All Fields]) OR "armed conflicts"[All Fields] OR "war"[All Fields]) AND "countries"[Title/Abstract]) OR "war-affected"[All Fields] OR "post-conflict"[All Fields] OR "post-war"[All Fields] OR "post-wars"[Title/Abstract] OR ("Middle East"[All Fields] OR "Middle East"[Title/Abstract] OR "eastern mediterranean"[Title/Abstract] OR "Afghanistan"[Title/Abstract] OR "Algeria"[Title/Abstract] OR "Djibouti"[Title/Abstract] OR "Egypt"[Title/Abstract] OR "Libya"[Title/Abstract] OR "morocco"[Title/Abstract] OR "Tunisia"[Title/Abstract] OR "Iran"[Title/Abstract] OR "Iraq"[Title/Abstract] OR "Jordan"[Title/Abstract] OR "Lebanon"[Title/Abstract] OR "Palestine"[Title/Abstract] OR "Gaza"[Title/Abstract] OR "Pakistan"[Title/Abstract] OR "Somalia"[Title/Abstract] OR "Somaliland"[Title/Abstract] OR "Sudan"[Title/Abstract] OR "Syria"[Title/Abstract] OR "syrian arab republic"[Title/Abstract] OR "west bank"[Title/Abstract] OR "Yemen"[Title/Abstract])) | 2,389 | 5:29:44 |
| 16 | #13 OR #14 | |  | "fragile state"[Title/Abstract] OR "fragile states"[Title/Abstract] OR ("fragile"[All Fields] AND "country"[Title/Abstract]) OR "fragile countries"[Title/Abstract] OR "fragile setting"[Title/Abstract] OR "fragile settings"[Title/Abstract] OR "fragile context"[Title/Abstract] OR "fragile contexts"[Title/Abstract] OR "weak state"[Title/Abstract] OR "conflict states"[Title/Abstract] OR "conflict state"[Title/Abstract] OR "conflict area"[Title/Abstract] OR "conflict areas"[Title/Abstract] OR "conflict country"[Title/Abstract] OR "conflict countries"[Title/Abstract] OR "conflict setting"[Title/Abstract] OR "conflict settings"[Title/Abstract] OR "conflict context"[Title/Abstract] OR "conflict contexts"[Title/Abstract] OR "conflict-affected"[Title/Abstract] OR "war setting"[Title/Abstract] OR "war settings"[Title/Abstract] OR "war context"[Title/Abstract] OR "war contexts"[Title/Abstract] OR (("armed conflicts"[MeSH Terms] OR ("armed"[All Fields] AND "conflicts"[All Fields]) OR "armed conflicts"[All Fields] OR "war"[All Fields]) AND "countries"[Title/Abstract]) OR "war-affected"[All Fields] OR "post-conflict"[All Fields] OR "post-war"[All Fields] OR "post-wars"[Title/Abstract] OR ("Middle East"[All Fields] OR "Middle East"[Title/Abstract] OR "eastern mediterranean"[Title/Abstract] OR "Afghanistan"[Title/Abstract] OR "Algeria"[Title/Abstract] OR "Djibouti"[Title/Abstract] OR "Egypt"[Title/Abstract] OR "Libya"[Title/Abstract] OR "morocco"[Title/Abstract] OR "Tunisia"[Title/Abstract] OR "Iran"[Title/Abstract] OR "Iraq"[Title/Abstract] OR "Jordan"[Title/Abstract] OR "Lebanon"[Title/Abstract] OR "Palestine"[Title/Abstract] OR "Gaza"[Title/Abstract] OR "Pakistan"[Title/Abstract] OR "Somalia"[Title/Abstract] OR "Somaliland"[Title/Abstract] OR "Sudan"[Title/Abstract] OR "Syria"[Title/Abstract] OR "syrian arab republic"[Title/Abstract] OR "west bank"[Title/Abstract] OR "Yemen"[Title/Abstract]) | 180,240 | 5:29:02 |
| 15 | #1 OR #2 OR #3 OR #4 OR #5 OR #6 OR #7 OR #8 OR #9 OR #10 OR #11 OR #12 | | | "Telemedicine"[MeSH Terms] OR "Telemedicine"[Title/Abstract] OR "telecare"[All Fields] OR "telehealth s"[All Fields] OR "Telemedicine"[MeSH Terms] OR "Telemedicine"[All Fields] OR "telehealth"[All Fields] OR "m-health"[All Fields] OR "mhealth s"[All Fields] OR "Telemedicine"[MeSH Terms] OR "Telemedicine"[All Fields] OR "mhealth"[All Fields] OR "e-health"[All Fields] OR "Telemedicine"[MeSH Terms] OR "Telemedicine"[All Fields] OR "ehealth"[All Fields] OR "interoperability"[All Fields] OR "interoperable"[All Fields] OR "interoperate"[All Fields] OR "interoperates"[All Fields] OR "interoperating"[All Fields] OR "interoperation"[All Fields] OR "digital health"[Title/Abstract] OR "digitized health"[Title/Abstract] OR "information system"[Title/Abstract] OR "information systems"[Title/Abstract] OR "information technology"[Title/Abstract] OR "information technologies"[Title/Abstract] OR "communication technology"[Title/Abstract] OR "cloud based system"[Title/Abstract] OR "cloud based systems"[Title/Abstract] OR "ICT"[Title/Abstract] OR "civil registration and vital statistics"[Title/Abstract] OR "electronic health"[Title/Abstract] OR "mobile health"[Title/Abstract] OR "health app"[Title/Abstract] OR "health apps"[Title/Abstract] OR "health application"[Title/Abstract] OR "mobile app"[Title/Abstract] OR "mobile apps"[Title/Abstract] OR "mobile application"[Title/Abstract] OR "mobile applications"[Title/Abstract] OR "health 2 0"[Title/Abstract] OR "medicine 2 0"[Title/Abstract] OR "data privacy"[Title/Abstract] OR "data security"[Title/Abstract] OR "data protection"[Title/Abstract] OR "data confidentiality"[Title/Abstract] OR "information privacy"[Title/Abstract] OR "information security"[Title/Abstract] OR "information protection"[Title/Abstract] OR "information confidentiality"[Title/Abstract] OR "electronic health record"[Title/Abstract] OR "electronic health records"[Title/Abstract] OR "electronic medical record"[Title/Abstract] OR "electronic medical records"[Title/Abstract] OR "health analytics"[Title/Abstract] OR "data analytics"[Title/Abstract] OR "big data"[Title/Abstract] | 207,918 | 5:28:26 |
| 14 | "Middle East"[exp:Mesh] OR middle east [tiab] OR Eastern Mediterranean[tiab] OR Afghanistan[tiab] OR Algeria[tiab] OR Djibouti[tiab] OR Egypt[tiab] OR Libya[tiab] OR morocco[tiab] OR Tunisia[tiab] OR Iran[tiab] OR Iraq[tiab] OR Jordan[tiab] OR Lebanon[tiab] OR Palestine [tiab] OR Gaza [tiab] OR Pakistan [tiab] OR Somalia [tiab] OR Somaliland[tiab] OR Sudan [tiab] OR Syria[tiab] OR syrian arab republic [tiab] OR west bank [tiab] OR Yemen[tiab] | | | "Middle East"[All Fields] OR "Middle East"[Title/Abstract] OR "eastern mediterranean"[Title/Abstract] OR "Afghanistan"[Title/Abstract] OR "Algeria"[Title/Abstract] OR "Djibouti"[Title/Abstract] OR "Egypt"[Title/Abstract] OR "Libya"[Title/Abstract] OR "morocco"[Title/Abstract] OR "Tunisia"[Title/Abstract] OR "Iran"[Title/Abstract] OR "Iraq"[Title/Abstract] OR "Jordan"[Title/Abstract] OR "Lebanon"[Title/Abstract] OR "Palestine"[Title/Abstract] OR "Gaza"[Title/Abstract] OR "Pakistan"[Title/Abstract] OR "Somalia"[Title/Abstract] OR "Somaliland"[Title/Abstract] OR "Sudan"[Title/Abstract] OR "Syria"[Title/Abstract] OR "syrian arab republic"[Title/Abstract] OR "west bank"[Title/Abstract] OR "Yemen"[Title/Abstract] | 173,288 | 5:25:26 |
| 13 | fragile state [tiab] OR fragile states [tiab] OR fragile country [tiab] OR fragile countries [tiab] OR fragile setting [tiab] OR fragile settings [tiab] OR fragile context [tiab] OR fragile contexts [tiab] OR weak state [tiab] OR conflict states [tiab] OR conflict state [tiab] OR conflict area [tiab] OR conflict areas [tiab] OR conflict country [tiab] OR conflict countries [tiab] OR conflict setting [tiab] OR conflict settings [tiab] OR conflict context [tiab] OR conflict contexts [tiab] OR conflict-affected [tiab] OR war setting [tiab] OR war settings [tiab] OR war context [tiab] OR war contexts [tiab] OR war countries [tiab] OR war-affected or post-conflict OR post-war OR post-wars [tiab] | | | "fragile state"[Title/Abstract] OR "fragile states"[Title/Abstract] OR ("fragile"[All Fields] AND "country"[Title/Abstract]) OR "fragile countries"[Title/Abstract] OR "fragile setting"[Title/Abstract] OR "fragile settings"[Title/Abstract] OR "fragile context"[Title/Abstract] OR "fragile contexts"[Title/Abstract] OR "weak state"[Title/Abstract] OR "conflict states"[Title/Abstract] OR "conflict state"[Title/Abstract] OR "conflict area"[Title/Abstract] OR "conflict areas"[Title/Abstract] OR "conflict country"[Title/Abstract] OR "conflict countries"[Title/Abstract] OR "conflict setting"[Title/Abstract] OR "conflict settings"[Title/Abstract] OR "conflict context"[Title/Abstract] OR "conflict contexts"[Title/Abstract] OR "conflict-affected"[Title/Abstract] OR "war setting"[Title/Abstract] OR "war settings"[Title/Abstract] OR "war context"[Title/Abstract] OR "war contexts"[Title/Abstract] OR (("armed conflicts"[MeSH Terms] OR ("armed"[All Fields] AND "conflicts"[All Fields]) OR "armed conflicts"[All Fields] OR "war"[All Fields]) AND "countries"[Title/Abstract]) OR "war-affected"[All Fields] OR "post-conflict"[All Fields] OR "post-war"[All Fields] OR "post-wars"[Title/Abstract] | 8,241 | 5:24:41 |
| 12 | health analytics [tiab] OR data analytics [tiab] OR big data [tiab] | | | "health analytics"[Title/Abstract] OR "data analytics"[Title/Abstract] OR "big data"[Title/Abstract] | 13,756 | 5:24:30 |
| 11 | electronic health record [tiab] OR electronic health records [tiab] OR electronic medical record[tiab] OR electronic medical records[tiab] | | | "electronic health record"[Title/Abstract] OR "electronic health records"[Title/Abstract] OR "electronic medical record"[Title/Abstract] OR "electronic medical records"[Title/Abstract] | 48,429 | 5:23:42 |
| 10 | data privacy [tiab] OR data security [tiab] OR data protection [tiab] OR data confidentiality [tiab] OR information privacy [tiab] OR information security [tiab] OR information protection [tiab] OR information confidentiality [tiab] | | | "data privacy"[Title/Abstract] OR "data security"[Title/Abstract] OR "data protection"[Title/Abstract] OR "data confidentiality"[Title/Abstract] OR "information privacy"[Title/Abstract] OR "information security"[Title/Abstract] OR "information protection"[Title/Abstract] OR "information confidentiality"[Title/Abstract] | 6,022 | 5:22:56 |
| 9 | health 2.0 [tiab] OR medicine 2.0 [tiab] | | | "health 2 0"[Title/Abstract] OR "medicine 2 0"[Title/Abstract] | 113 | 5:22:27 |
| 8 | health app[tiab] OR health apps[tiab] OR health application[tiab] OR mobile app[tiab] OR mobile apps[tiab] OR mobile application[tiab] OR mobile applications[tiab] | | | "health app"[Title/Abstract] OR "health apps"[Title/Abstract] OR "health application"[Title/Abstract] OR "mobile app"[Title/Abstract] OR "mobile apps"[Title/Abstract] OR "mobile application"[Title/Abstract] OR "mobile applications"[Title/Abstract] | 10,022 | 5:22:13 |
| 7 | electronic health [tiab] OR mobile health [tiab] | | | "electronic health"[Title/Abstract] OR "mobile health"[Title/Abstract] | 34,946 | 5:21:47 |
| 6 | civil registration and vital statistics [tiab] | | | "civil registration and vital statistics"[Title/Abstract] | 130 | 5:21:37 |
| 5 | ICT [tiab] |  |  | "ICT"[Title/Abstract] | 7,245 | 5:21:26 |
| 4 | cloud-based system [tiab] OR cloud-based systems [tiab] | | | "cloud based system"[Title/Abstract] OR "cloud based systems"[Title/Abstract] | 79 | 5:21:03 |
| 3 | information system [tiab] OR information systems [tiab] OR information technology [tiab] OR information technologies [tiab] OR communication technology [tiab] | | | "information system"[Title/Abstract] OR "information systems"[Title/Abstract] OR "information technology"[Title/Abstract] OR "information technologies"[Title/Abstract] OR "communication technology"[Title/Abstract] | 57,667 | 5:20:49 |
| 2 | Digital health [tiab] OR Digitized health [tiab] | | | "digital health"[Title/Abstract] OR "digitized health"[Title/Abstract] | 5,936 | 5:20:29 |
| 1 | telemedicine[MeSH Terms] OR Telemedicine[tiab] OR telecare OR telehealth OR m-health OR mhealth OR e-health OR ehealth OR Interoperability | | | "Telemedicine"[MeSH Terms] OR "Telemedicine"[Title/Abstract] OR "telecare"[All Fields] OR "telehealth s"[All Fields] OR "Telemedicine"[MeSH Terms] OR "Telemedicine"[All Fields] OR "telehealth"[All Fields] OR "m-health"[All Fields] OR "mhealth s"[All Fields] OR "Telemedicine"[MeSH Terms] OR "Telemedicine"[All Fields] OR "mhealth"[All Fields] OR "e-health"[All Fields] OR "Telemedicine"[MeSH Terms] OR "Telemedicine"[All Fields] OR "ehealth"[All Fields] OR "interoperability"[All Fields] OR "interoperable"[All Fields] OR "interoperate"[All Fields] OR "interoperates"[All Fields] OR "interoperating"[All Fields] OR "interoperation"[All Fields] | 81,138 | 5:19:50 |

| Recent queries in pubmed | |  |  |
| --- | --- | --- | --- |
| Search | Query | Items found | Time |
| #50 | Search #47 AND #48 | 1296 | 10:39:52 |
| #48 | Search #43 OR #45 | 129044 | 10:38:16 |
| #47 | Search #13 OR #14 OR #16 OR #18 OR #19 OR #20 OR #23 OR #30 OR #32 OR #33 OR #34 OR #46 | 116247 | 10:37:29 |
| #46 | Search health analytics [tiab] OR data analytics [tiab] OR big data [tiab] | 5242 | 10:35:08 |
| #45 | Search fragile state [tiab] OR fragile states [tiab] OR fragile country [tiab] OR fragile countries [tiab] OR fragile setting [tiab] OR fragile settings [tiab] OR fragile context [tiab] OR fragile contexts [tiab] OR weak state [tiab] OR conflict states [tiab] OR conflict state [tiab] OR conflict area [tiab] OR conflict areas [tiab] OR conflict country [tiab] OR conflict countries [tiab] OR conflict setting [tiab] OR conflict settings [tiab] OR conflict context [tiab] OR conflict contexts [tiab] OR conflict-affected [tiab] OR war setting [tiab] OR war settings [tiab] OR war context [tiab] OR war contexts [tiab] OR war countries [tiab] OR war-affected or post-conflict OR post-war OR post-wars [tiab] | 13850 | 10:29:45 |
| #43 | Search "Middle East"[exp:Mesh] OR middle east [tiab] OR Eastern Mediterranean[tiab] OR Afghanistan[tiab] OR Algeria[tiab] OR Djibouti[tiab] OR Egypt[tiab] OR Libya[tiab] OR morocco[tiab] OR Tunisia[tiab] OR Iran[tiab] OR Iraq[tiab] OR Jordan[tiab] OR Lebanon[tiab] OR Palestine [tiab] OR Gaza [tiab] OR Pakistan [tiab] OR Somalia [tiab] OR Somaliland[tiab] OR Sudan [tiab] OR Syria[tiab] OR syrian arab republic [tiab] OR west bank [tiab] OR Yemen[tiab] | 116275 | 10:19:14 |
| #34 | Search electronic health record [tiab] OR electronic health records [tiab] OR electronic medical record[tiab] OR electronic medical records[tiab] | 24956 | 10:10:16 |
| #33 | Search data privacy [tiab] OR data security [tiab] OR data protection [tiab] OR data confidentiality [tiab] OR information privacy [tiab] OR information security [tiab] OR information protection [tiab] OR information confidentiality [tiab] | 3052 | 10:08:45 |
| #32 | Search health 2.0 [tiab] OR medicine 2.0 [tiab] | 94 | 10:06:37 |
| #30 | Search health app[tiab] OR health apps[tiab] OR health application[tiab] OR mobile app[tiab] OR mobile apps[tiab] OR mobile application[tiab] OR mobile applications[tiab] | 3542 | 9:59:49 |
| #23 | Search electronic health [tiab] OR mobile health [tiab] | 16134 | 9:41:20 |
| #20 | Search civil registration and vital statistics [tiab] | 50 | 9:40:20 |
| #19 | Search ICT [tiab] | 4697 | 9:39:55 |
| #18 | Search cloud-based system [tiab] OR cloud-based systems [tiab] | 37 | 9:39:29 |
| #16 | Search information system [tiab] OR information systems [tiab] OR information technology [tiab] OR information technologies [tiab] OR communication technology [tiab] | 41467 | 9:29:47 |
| #14 | Search Digital health [tiab] OR Digitized health [tiab] | 1145 | 9:27:05 |
| #13 | Search telemedicine[MeSH Terms] OR Telemedicine[tiab] OR telecare OR telehealth OR m-health OR mhealth OR e-health OR ehealth OR Interoperability | 42414 | 9:26:16 |

IDRC Databases Search Strategy

|  | Thursday, February 07, 2019 7:33:06 AM |
| --- | --- |

| **Search ID#** | **Search Terms** | **Search Options** | **Last Run Via** | **Results** |
| --- | --- | --- | --- | --- |
| S21 | S19 AND S20 | Expanders - Apply related words  Search modes - Boolean/Phrase | Interface - EBSCO Discovery Service  Search Screen - Advanced Search  Database - Electronic Collections / Collections électroniques | Display |
| S20 | S13 OR S14 OR S15 OR S16 OR S17 OR S18 | Expanders - Apply related words  Search modes - Boolean/Phrase | Interface - EBSCO Discovery Service  Search Screen - Advanced Search  Database - Electronic Collections / Collections électroniques | Display |
| S19 | S1 OR S2 OR S3 OR S4 OR S5 OR S6 OR S7 OR S8 OR S9 OR S10 OR S11 OR S12 | Expanders - Apply related words  Search modes - Boolean/Phrase | Interface - EBSCO Discovery Service  Search Screen - Advanced Search  Database - Electronic Collections / Collections électroniques | Display |
| S18 | TI (middle east) N1 (north* Africa) OR AB (middle east) N1 (north* Africa) OR SU (middle east) N1 (north* Africa) | Expanders - Apply related words  Search modes - Boolean/Phrase | Interface - EBSCO Discovery Service  Search Screen - Advanced Search  Database - Electronic Collections / Collections électroniques | Display |
| S17 | TI ( (fragile or weak or fragility or conflict or conflicts or conflict-affected or war or wars or war-affected or post-conflict* or post-war or post-wars) N1 (setting* or context or contexts or state or states or country or countries) ) OR AB ( (fragile or weak or fragility or conflict or conflicts or conflict-affected or war or wars or war-affected or post-conflict* or post-war or post-wars) N1 (setting* or context or contexts or state or states or country or countries) ) OR SU ( (fragile or weak or fragility or conflict or conflicts or conflict-affected or war or wars or war-affected or post-conflict* or post-war or post-wars) N1 (setting* or context or contexts or state or states or country or countries) ) | Expanders - Apply related words  Search modes - Boolean/Phrase | Interface - EBSCO Discovery Service  Search Screen - Advanced Search  Database - Electronic Collections / Collections électroniques | Display |
| S16 | TI East* N0 Mediterranean OR AB East* N0 Mediterranean OR SU East* N0 Mediterranean | Expanders - Apply related words  Search modes - Boolean/Phrase | Interface - EBSCO Discovery Service  Search Screen - Advanced Search  Database - Electronic Collections / Collections électroniques | Display |
| S15 | TI (syrian arab* republic) OR AB (syrian arab* republic) OR SU (syrian arab* republic) | Limiters - Date Published: 20000101-20191231  Expanders - Apply related words  Search modes - Boolean/Phrase | Interface - EBSCO Discovery Service  Search Screen - Advanced Search  Database - Electronic Collections / Collections électroniques | Display |
| S14 | TI West N1 bank OR AB West N1 bank OR SU West N1 bank | Limiters - Date Published: 20000101-20191231  Expanders - Apply related words  Search modes - Boolean/Phrase | Interface - EBSCO Discovery Service  Search Screen - Advanced Search  Database - Electronic Collections / Collections électroniques | Display |
| S13 | TI ( Afghanistan or Algeria or Djibouti or Egypt or Libya or morocco or Tunisia or Iran or Iraq or Jordan or Lebanon or Palestine or Gaza or Pakistan or Somalia or Somaliland or Sudan or Syria or Yemen ) OR AB ( Afghanistan or Algeria or Djibouti or Egypt or Libya or morocco or Tunisia or Iran or Iraq or Jordan or Lebanon or Palestine or Gaza or Pakistan or Somalia or Somaliland or Sudan or Syria or Yemen ) OR KW( Afghanistan or Algeria or Djibouti or Egypt or Libya or morocco or Tunisia or Iran or Iraq or Jordan or Lebanon or Palestine or Gaza or Pakistan or Somalia or Somaliland or Sudan or Syria or Yemen ) | Limiters - Date Published: 20000101-20191231  Expanders - Apply related words  Search modes - Boolean/Phrase | Interface - EBSCO Discovery Service  Search Screen - Advanced Search  Database - Electronic Collections / Collections électroniques | Display |
| S12 | TI ( electronic N2 (record or records) ) OR AB ( electronic N2 (record or records) ) OR SU ( electronic N2 (record or records) ) | Limiters - Date Published: 20000101-20191231  Expanders - Apply related words  Search modes - Boolean/Phrase | Interface - EBSCO Discovery Service  Search Screen - Advanced Search  Database - Electronic Collections / Collections électroniques | Display |
| S11 | TI "medicine 2.0" OR AB "medicine 2.0" OR SU "medicine 2.0" | Limiters - Date Published: 20000101-20191231  Expanders - Apply related words  Search modes - Boolean/Phrase | Interface - EBSCO Discovery Service  Search Screen - Advanced Search  Database - Electronic Collections / Collections électroniques | Display |
| S10 | TI ( (Privacy or security or protection or confidentiality) N2 (data or information or internet) ) OR AB ( (Privacy or security or protection or confidentiality) N2 (data or information or internet) ) OR SU ( (Privacy or security or protection or confidentiality) N2 (data or information or internet) ) | Limiters - Date Published: 20000101-20191231  Expanders - Apply related words  Search modes - Boolean/Phrase | Interface - EBSCO Discovery Service  Search Screen - Advanced Search  Database - Electronic Collections / Collections électroniques | Display |
| S9 | TI health N1 analytics OR AB health N1 analytics OR SU health N1 analytics | Limiters - Date Published: 20000101-20191231  Expanders - Apply related words  Search modes - Boolean/Phrase | Interface - EBSCO Discovery Service  Search Screen - Advanced Search  Database - Electronic Collections / Collections électroniques | Display |
| S8 | TI "big data" OR AB "big data" OR SU "big data" | Limiters - Date Published: 20000101-20191231  Expanders - Apply related words  Search modes - Boolean/Phrase | Interface - EBSCO Discovery Service  Search Screen - Advanced Search  Database - Electronic Collections / Collections électroniques | Display |
| S7 | TI "health 2.0" OR AB "health 2.0" OR SU "health 2.0" | Limiters - Date Published: 20000101-20191231  Expanders - Apply related words  Search modes - Boolean/Phrase | Interface - EBSCO Discovery Service  Search Screen - Advanced Search  Database - Electronic Collections / Collections électroniques | Display |
| S6 | TI ( (health or wellness or mobile) N1 (app or apps or application or applications) ) OR AB ( (health or wellness or mobile) N1 (app or apps or application or applications) ) OR SU ( (health or wellness or mobile) N1 (app or apps or application or applications) ) | Limiters - Date Published: 20000101-20191231  Expanders - Apply related words  Search modes - Boolean/Phrase | Interface - EBSCO Discovery Service  Search Screen - Advanced Search  Database - Electronic Collections / Collections électroniques | Display |
| S5 | TI ( (electronic or mobile) N1 health ) OR AB ( (electronic or mobile) N1 health ) OR SU ( (electronic or mobile) N1 health ) | Limiters - Date Published: 20000101-20191231  Expanders - Apply related words  Search modes - Boolean/Phrase | Interface - EBSCO Discovery Service  Search Screen - Advanced Search  Database - Electronic Collections / Collections électroniques | Display |
| S4 | TI ( civil registration and vital statistics ) OR AB ( civil registration and vital statistics ) OR SU ( civil registration and vital statistics ) | Limiters - Date Published: 20000101-20191231  Expanders - Apply related words  Search modes - Boolean/Phrase | Interface - EBSCO Discovery Service  Search Screen - Advanced Search  Database - Electronic Collections / Collections électroniques | Display |
| S3 | TI (information N1 (system or systems or technology) ) OR AB (information N1 (system or systems or technology) ) OR KW(information N1 (system or systems or technology) ) | Limiters - Date Published: 20000101-20201231  Expanders - Apply related words  Search modes - Boolean/Phrase | Interface - EBSCO Discovery Service  Search Screen - Advanced Search  Database - Electronic Collections / Collections électroniques | Display |
| S2 | TI ( (Digital or Digitized) N2 health ) OR AB ( (Digital or Digitized) N2 health ) OR SU ( (Digital or Digitized) N2 health ) | Limiters - Date Published: 20000101-20191231  Expanders - Apply related words  Search modes - Boolean/Phrase | Interface - EBSCO Discovery Service  Search Screen - Advanced Search  Database - Electronic Collections / Collections électroniques | Display |
| S1 | TI ( telemedicine or telehealth or telecare or e-health or ehealth or m-health or mhealth or e-mental or cloud-based or Interoperability ) OR AB ( telemedicine or telehealth or telecare or e-health or ehealth or m-health or mhealth or e-mental or cloud-based or Interoperability ) OR SU ( telemedicine or telehealth or telecare or e-health or ehealth or m-health or mhealth or e-mental or cloud-based or Interoperability ) | Limiters - Date Published: 20000101-20201231  Expanders - Apply related words  Search modes - Boolean/Phrase | Interface - EBSCO Discovery Service  Search Screen - Advanced Search  Database - Electronic Collections / Collections électroniques | Display |

Health Systems Evidence Search Strategy

HSE databases searched on Feb 4, 2019 and update on October 2022

(Afghanistan OR Algeria OR Djibouti OR Egypt OR Libya OR morocco OR Tunisia OR Iran OR Iraq OR Jordan OR Lebanon OR Palestine OR Gaza OR Pakistan OR Somalia OR Sudan OR Syria OR (west bank) OR Yemen OR (middle AND east*) OR (north* AND Africa) OR (East* AND Mediterranean) OR Fragile OR fragility OR conflict OR conflicts OR conflict-affected OR war OR wars OR war-affected OR post-conflict OR post-conflicts OR post-war OR post-wars) **AND** ((Digital health) OR (Digitized health) OR cloud-based OR ICT OR (information system) OR (information systems) OR (information technology) OR (civil registration vital statistics) OR (electronic health) OR (mobile health) OR m-health OR mhealth OR e-health OR ehealth OR “health app” OR “health apps” OR “health application” OR “health applications” OR “wellness app” OR “wellness apps” OR “wellness application” OR “wellness applications” OR “mobile app” OR “mobile apps” OR “mobile application” OR “mobile applications” OR “health 2.0” OR “medicine 2.0” OR Privacy OR security OR protection OR confidentiality OR (electronic record) OR (medical record) OR (electronic records) OR (medical records) OR Telemedicine OR telecare OR telehealth OR Interoperability OR (big data) OR (health analytics))

Index Medicus for the Eastern Mediterranean Region

Digital health (109):  <https://vlibrary.emro.who.int/searchd/page/2/?skeyword=Digital%20health&journal_title&fauthor_title%5B0%5D&mesh_title%5B0%5D&relation&index_option&format=summary&sort=PublicationDate&perpage=10&adv&database=imemr&syear=range&year_from=2020&year_to=2022&records=>

Digitalized health (5): <https://vlibrary.emro.who.int/searchd/?skeyword=digitalized+health&index_option=&journal_title=&fauthor_title%5B%5D=&mesh_title%5B%5D=&database=imemr&journal=&country=&syear=&typepublication=&fulltextlanguage=&abstractlanguage=&geographic=&category=&year_from=&year_to=&relation=&rc_verification_id=&who_subject=&format=summary&sort=PublicationDate&perpage=200&records=>

Digitized health: No Results Found

mobile health (108): <https://vlibrary.emro.who.int/searchd/page/2/?skeyword=mobile%20health&journal_title&fauthor_title%5B0%5D&mesh_title%5B0%5D&relation&index_option&format=summary&sort=PublicationDate&perpage=10&adv&database=imemr&syear=range&year_from=2020&year_to=2022&records=>

m-health: No Results Found

mhealth (12): <https://vlibrary.emro.who.int/searchd/page/2/?skeyword=mhealth&format=summary&sort=PublicationDate&perpage=10&database=imemr&journal&journal_title&fauthor_title%5B0%5D&mesh_title%5B0%5D&country&syear&typepublication&fulltextlanguage&abstractlanguage&geographic&category&year_from&adv&year_to&rc_verification_id&who_subject&records=>

e-health (26): <https://vlibrary.emro.who.int/searchd/page/2/?skeyword=e-health&journal_title&fauthor_title%5B0%5D&mesh_title%5B0%5D&relation&index_option&format=summary&sort=PublicationDate&perpage=10&adv&database=imemr&syear=range&year_from=2020&year_to=2022&records=>

ehealth (24): <https://vlibrary.emro.who.int/searchd/page/2/?skeyword=ehealth&journal_title&fauthor_title%5B0%5D&mesh_title%5B0%5D&relation&index_option&format=summary&sort=PublicationDate&perpage=10&adv&database=imemr&syear=range&year_from=2020&year_to=2022&records=>

Telemedicine (88): <https://vlibrary.emro.who.int/searchd/page/2/?skeyword=Telemedicine&journal_title&fauthor_title%5B0%5D&mesh_title%5B0%5D&relation&index_option&format=summary&sort=PublicationDate&perpage=10&adv&database=imemr&syear=range&year_from=2020&year_to=2022&records=>

telecare: No Results Found

telehealth (19): <https://vlibrary.emro.who.int/searchd/page/2/?skeyword=telehealth&journal_title&fauthor_title%5B0%5D&mesh_title%5B0%5D&relation&index_option&format=summary&sort=PublicationDate&perpage=10&adv&database=imemr&syear=range&year_from=2020&year_to=2022&records=>

big data (112): <https://vlibrary.emro.who.int/searchd/page/2/?skeyword=big%20data&journal_title&fauthor_title%5B0%5D&mesh_title%5B0%5D&relation&index_option&format=summary&sort=PublicationDate&perpage=10&adv&database=imemr&syear=range&year_from=2020&year_to=2022&records=>

CENTRAL Search Strategy

Update to August 2022 identified 107 articles

Search Name: digital health

Last Saved: 07/02/2019 16:04:09

Comment:

ID Search

#1 MeSH descriptor: [Medical Records Systems, Computerized] explode all trees

#2 MeSH descriptor: [Information Science] explode all trees

#3 (telemedicine or telehealth or telecare or e-health or ehealth or m-health or mhealth or e-mental or cloud-based or Interoperability):ti,ab,kw (Word variations have been searched)

#4 ((Digital OR Digitized) Next/2 health):ti,ab,kw

#5 (information Next/2 (system or systems or technology or technologies)):ti,ab,kw

#6 ((civil AND registration) Near/1 (vital AND statistics)):ti,ab,kw

#7 ((electronic OR mobile) Next/2 health):ti,ab,kw

#8 ((health OR wellness OR mobile) Near/2 (app OR apps OR application OR applications)):ti,ab,kw

#9 ("health 2.0" OR "medicine 2.0"):ti,ab,kw

#10 ("big data"):ti,ab,kw

#11 (health Near/1 analytics):ti,ab,kw

#12 ((Privacy or security or protection or confidentiality) Near/2 (data or information or internet)):ti,ab,kw

#13 (electronic Near/2 (record or records)):ti,ab,kw

#14 (Afghanistan or Algeria or Djibouti or Egypt or Libya or morocco or Tunisia or Iran or Iraq or Jordan or Lebanon or Palestine or Gaza or Pakistan or Somalia or Somaliland or Sudan or Syria or Yemen):ti,ab,kw

#15 ("West bank"):ti,ab,kw

#16 ("syrian arab* republic"):ti,ab,kw

#17 (East* Near/1 Mediterranean):ti,ab,kw

#18 ((middle east) Near/1 (north* Africa)):ti,ab,kw

#19 (middle Near/1 east*):ti,ab,kw

#20 (north* Near/1 Africa):ti,ab,kw

#21 ((fragile or weak or fragility or conflict or conflicts or conflict-affected or war or wars or war-affected or post-conflict* or post-war or post-wars) Near/2 (setting* or context or contexts or areas or state or states or country or countries)):ti,ab,kw

#22 #1 OR #2 OR #3 OR #4 OR #5 OR #6 OR #7 OR #8 Or #9 OR #10 OR #11 OR #12 OR

#23 #14 OR #15 OR #16 OR #17 OR #18 OR #19 OR #20 OR #21

#24 #22 AND #23
