# Supplementary material for: Digital health in fragile states in the Middle East and North Africa (MENA) region: A scoping review of the literature
Source: PLoS One. 2023 Apr 28;18(4):e0285226. doi: 10.1371/journal.pone.0285226 (PMC10146476; doi:10.1371/journal.pone.0285226)
Supplement: S2 Appendix — (DOCX) [file pone.0285226.s003.docx]

S2 Appendix: List of excluded articles with reason for exclusion

| Citation | Reason for Exclusion |
| --- | --- |
| (2004). "news in brief." Nature 431(7009): 620-620. | Not Design of Interest |
| Ababtain, A. F., et al. (2013). "The state of mobile health in the developing world and the Middle East." Studies in Health Technology & Informatics 190: 300-302. | Not country of Interest |
| Abd Ghani, M. K., et al. (2008). "A flexible telemedicine framework for the continuous upkeep of patient lifetime health records (F2U-LHR)." AMCIS 2008 Proceedings: 110. | Not country of Interest |
| Abu-El-Noor, N. I., et al. (2020). "Impact of a mobile phone app on adherence to treatment regimens among hypertensive patients: A randomised clinical trial study." European journal of cardiovascular nursing : journal of the Working Group on Cardiovascular Nursing of the European Society of Cardiology: 1474515120938235. | Duplicate |
| Acharibasam, J. W. and R. Wynn (2018). "Telemental health in low-and middle-income countries: A systematic review." International journal of telemedicine and applications 2018. | Not country of Interest |
| Ahmed, A. M., et al. (2008). "Use of the Internet by Sudanese doctors and medical students." Eastern Mediterranean Health Journal 14(1): 134-141. | Not Timeframe of Interest |
| Alajlani, M. (2010). Issues facing the application of telemedicine in developing countries: Hashemite Kingdom of Jordan and Syrian Arab Republic, Brunel University, School of Information Systems, Computing and Mathematics. | Not country of Interest |
| Alajlani, M. and M. Clarke (2013). "Effect of culture on acceptance of telemedicine in Middle Eastern countries: case study of Jordan and Syria." Telemedicine and e-Health 19(4): 305-311. | Not country of Interest |
| Alghamdi, M., et al. (2015). "A systematic review of mobile health technology use in developing countries." Studies in health technology and informatics 213: 223-226. | Not country of Interest |
| Ali, F. R., et al. (2012). "Letter: Real-time, intercontinental dermatology teaching of trainee physicians in Somaliland using a dedicated social networking portal." Dermatology Online Journal 18(4): 16. | Not Design of Interest |
| Anthony, C. R., et al. (2018). "Health Sector Reform in the Kurdistan Region-Iraq: Primary Care Management Information System, Physician Dual Practice Finance Reform, and Quality of Care Training." Rand Health Quarterly 8(2): 1. | Not Topic of Interest |
| Anwar, F., et al. (2011). "Barriers in adoption of health information technology in developing societies." Int J Adv Comput Sci Appl 2(8): 40-45. | Not country of Interest |
| Arabi, M., et al. (2017). "Practice of teleradiology in crisis zones: the unique case of Syria." The Lancet Global Health 5(4): e399-e400. | Not Design of Interest |
| Asi, Y. M. and C. Williams (2018). "The role of digital health in making progress toward Sustainable Development Goal (SDG) 3 in conflict-affected populations." International Journal of Medical Informatics 114: 114-120. | Not country of Interest |
| Azizy, A., et al. (2020). "Do Not Forget Afghanistan in Times of COVID-19: Telemedicine and the Internet of Things to Strengthen Planetary Health Systems." Omics a Journal of Integrative Biology 24(6): 311-313. | Not Design of Interest |
| Bardus;, M., et al. (2017). "A Self-directed Mobile Intervention to Promote Weight Control Among Employees of a Lebanese University." Https://clinicaltrials.gov/show/nct03321331. | Not Design of Interest |
| Ba-Saddik, I. A. (2013). "Childhood cancer in Aden, Yemen." Cancer epidemiology 37(6): 803-806. | Not Topic of Interest |
| Bhutta, Z. A. (2020). "Reaching the unreached; mobile health teams in conflict settings." Archives of Disease in Childhood 105(1): 4-5. | Not Topic of Interest |
| Bolton, P., et al. (2014). "A randomized controlled trial of mental health interventions for survivors of systematic violence in Kurdistan, Northern Iraq." BMC psychiatry 14(1): 360. | Not Topic of Interest |
| Bragg, M. A., et al. (2011). "Research forum abstract: 346 Implementing an Emergency Medicine Health Information System in Baghdad, Iraq." Annals of Emergency Medicine 58(Supplement): S294. | Not Topic of Interest |
| Caffery, L., et al. (2005). "Telemedical support for Iraq." Journal of Telemedicine & Telecare 11: 110-110. | Not Design of Interest |
| Chen, H., et al. (2018). "Effectiveness and appropriateness of mHealth interventions for maternal and child health: systematic review." JMIR MHealth and UHealth 6(1): e7. | Not country of Interest |
| Cummings, S. and M. Rowbotham (2017). "Electronic informed consent and internet-based trials." New England Journal of Medicine 376(9): 859-861. | Not Topic of Interest |
| Daher, R. and H. Awada (2007). "[The usefulness of personal digital assistants (Palm and Pocket PC) in the medical field]." Journal Medical Libanais - Lebanese Medical Journal 55(1): 19-28. | Not Timeframe of Interest |
| Dailey, J. I. and M. R. Stanfa-Brew (2014). "Telebehavioral health in Afghanistan." Military Medicine 179(7): 708-710. | Not Topic of Interest |
| Danis, C. M., et al. (2010). Mobile phones for health education in the developing world: SMS as a user interface. Proceedings of the First ACM Symposium on Computing for Development. | Not country of Interest |
| Decosas, J. and L. Mbuagbaw (2018). "Networking in eHealth research: results of the IDRC SEARCH program evaluation." Journal of Public Health 40(suppl_2): ii12-ii15. | Not country of Interest |
| Dott, M. M., et al. (2005). "Implementing a facility-based maternal and perinatal health care surveillance system in Afghanistan." Journal of Midwifery & Women's Health 50(4): 296-300. | Not Topic of Interest |
| El Gatit, A. M., et al. (2008). "Effects of an awareness symposium on perception of Libyan physicians regarding telemedicine." Eastern Mediterranean Health Journal 14(4): 926-930. | Not Timeframe of Interest |
| El Oakley, R. M., et al. (2013). "Consultation on the Libyan health systems: towards patient-centred services." Libyan Journal of Medicine 8. | Not Topic of Interest |
| Feroz, A., et al. (2018). "Health systems readiness for adopting mhealth interventions for addressing non-communicable diseases in low-and middle-income countries: a current debate." Global health action 11(1): 1496887. | Not country of Interest |
| Free, C., et al. (2013). "The effectiveness of mobile-health technology-based health behaviour change or disease management interventions for health care consumers: a systematic review." PLoS med 10(1): e1001362. | Not country of Interest |
| Friberg, I. K., et al. (2019). "Antenatal care data sources and their policy and planning implications: a Palestinian example using the Lives Saved Tool." BMC Public Health 19(1): 124-124. | Not Topic of Interest |
| Frøen, J. F., et al. (2016). "eRegistries: Electronic registries for maternal and child health." BMC Pregnancy and Childbirth 16(1): 11. | Not country of Interest |
| Gonçalves‐Bradley, D. C., et al. (2018). "Mobile‐based technologies to support client to healthcare provider communication and management of care." The Cochrane Database of Systematic Reviews 2018(1). | Not Design of Interest |
| H , Tornqvist. (2000). What Are the Barriers Facing the Telemedicine, A Report from the Project on “Telemedicine– Regional and National Collaboration” Subproject: “Incentives and Implementation”’. | Not country of Interest |
| Hilty, D. M., et al. (2013). "The effectiveness of telemental health: a 2013 review." Telemedicine and e-Health 19(6): 444-454. | Not country of Interest |
| Househ, M. (2012). "Mobile social networking health (MSNet-health): Beyond the mHealth frontier." Studies in Health Technology & Informatics 180: 808-812. | Not country of Interest |
| Househ, M., et al. (2012). mHealth: a passing fad or here to stay? Telemedicine and e-health services, policies, and applications: Advancements and developments, IGI Global: 151-178.. | Not Design of Interest |
| Ismail, A., et al. (2018). "Store‐and‐forward teledermatology service for primary care providers in Afghanistan." International Journal of Dermatology 57(11): e145-e147. | Not Design of Interest |
| Iversen, B. G., et al. (2013). "Abstracts: Assessment of the cause of death registry in the occupied Palestinian territory: a qualitative study." The Lancet 382(Supplement 4): S21. | Not Topic of Interest |
| Iyawa, G. E., et al. (2016). "Digital health innovation ecosystems: From systematic literature review to conceptual framework." | Not country of Interest |
| Jackson, D., et al. (2018). "Civil registration and vital statistics in health systems." Bulletin of the World Health Organization 96(12): 861. | Not country of Interest |
| Jasem, J. A., et al. (2014). "An epidemiological analysis of acute flaccid paralysis and its surveillance system in Iraq, 1997-2011." BMC Infectious Diseases 14: 448. | Not Topic of Interest |
| Jefee-Bahloul, H. (2014). "Use of telepsychiatry in areas of conflict: the Syrian refugee crisis as an example." Journal of Telemedicine & Telecare 20(3): 167-168. | Not country of Interest |
| Jefee-Bahloul, H., et al. (2014). "Pilot assessment and survey of Syrian refugees' psychological stress and openness to referral for telepsychiatry (PASSPORT Study)." Telemedicine Journal & E-Health 20(10): 977-979. | Not country of Interest |
| Jefee-Bahloul, H., et al. (2016). "Using a Store-and-Forward System to Provide Global Telemental Health Supervision and Training: A Case from Syria." Academic Psychiatry 40(4): 707-709. | Not Design of Interest |
| Kahn, J. G., et al. (2010). "‘Mobile’health needs and opportunities in developing countries." Health affairs 29(2): 252-258. | Not country of Interest |
| Kay, M., et al. (2011). "mHealth: New horizons for health through mobile technologies." World Health Organization 64(7): 66-71. | Not country of Interest |
| Knaevelsrud, C., et al. (2015). "Web-based psychotherapy for posttraumatic stress disorder in war-traumatized Arab patients: randomized controlled trial." Journal of medical internet research 17(3): e71. | Not country of Interest |
| Kokia, E. S., et al. (2006). "The use of medical informatics as a management tool for community health services during the 2006 Israel-Lebanon War." Israel Medical Association Journal: Imaj 8(12): 865-869. | Not country of Interest |
| Kurdy, M. B. (2006). Competencies and training needs in the IST in SYRIA and Mediterranean Countries. 2006 2nd International conference on information & communication technologies, IEEE. | Not country of Interest |
| Lami, F., et al. (2019). "Real-Time Surveillance of Infectious Diseases and Other Health Conditions During Iraq's Arbaeenia Mass Gathering: Cross-Sectional Study." JMIR public health and surveillance 5(4): e14510. | Not Topic of Interest |
| Lee, S. H., et al. (2016). "Effectiveness of mHealth interventions for maternal, newborn and child health in low–and middle–income countries: Systematic review and meta–analysis." Journal of global health 6(1). | Not country of Interest |
| Lemaire, J. (2011). Scaling up mobile health elements necessary for the mHealth in developing countries, Actevis Consulting Group. Retrieved from https://www. k4health. org/sites …. | Not country of Interest |
| Majed Kamel, A.-A., et al. (2019). "MHealth for Decision Making Support: A Case Study of EHealth in the Public Sector." | Not country of Interest |
| Marcolino, M. S., et al. (2018). "The impact of mHealth interventions: systematic review of systematic reviews." JMIR MHealth and UHealth 6(1): e23. | Not country of Interest |
| Marini, S. D. (2000). "Introduction of nursing informatics in the nursing baccalaureate program at the American University of Beirut." Computers in Nursing 18(5): 240-247. | Not Topic of Interest |
| Marini, S. D., et al. (2009). "Information technology for medication administration: assessing bedside readiness among nurses in Lebanon." International Journal of Evidence-Based Healthcare 7(1): 49-58. | Not Timeframe of Interest |
| McCarthy, O. L., et al. (2018). "Development of an intervention delivered by mobile phone aimed at decreasing unintended pregnancy among young people in three lower middle income countries." BMC Public Health 18(1): 576. | Not country of Interest |
| Merrell, R. C. and C. R. Doarn (2016). "Tales of Telemedicine in Syria." Telemedicine Journal & E-Health 22(12): 969-970. | Not Design of Interest |
| Mesmar, S., et al. (2016). "The impact of digital technology on health of populations affected by humanitarian crises: Recent innovations and current gaps." Journal of public health policy 37(2): 167-200. | Not country of Interest |
| Miller, K. E. and A. Rasmussen (2010). "War exposure, daily stressors, and mental health in conflict and post-conflict settings: bridging the divide between trauma-focused and psychosocial frameworks." Social science & medicine 70(1): 7-16. | Not Topic of Interest |
| Mohammed, M. A., et al. (2012). E-government architecture uses data warehouse techniques to increase information sharing in Iraqi universities. 2012 IEEE Symposium on E-Learning, E-Management and E-Services, IEEE. | Not Topic of Interest |
| Moja, L., et al. (2014). "Effectiveness of computerized decision support systems linked to electronic health records: a systematic review and meta-analysis." American journal of public health 104(12): e12-e22. | Not country of Interest |
| Moughrabieh, A. and C. Weinert (2016). "Rapid Deployment of International Tele-Intensive Care Unit Services in War-Torn Syria." Annals of the American Thoracic Society 13(2): 165-172. | Not Design of Interest |
| Mousavi, S. H., et al. (2020). "Mapping the Changes on Incidence, Case Fatality Rates and Recovery Proportion of COVID-19 in Afghanistan Using Geographical Information Systems." Archives of Medical Research 18: 18. | Not Design of Interest |
| Muhjazi, G., et al. (2013). "An early warning and response system for Syria." Lancet 382(9910): 2066. | Not Design of Interest |
| Mulvaney, D., et al. (2012). "Development of m-health monitoring systems in India and Iraq." Conference Proceedings: ... Annual International Conference of the IEEE Engineering in Medicine & Biology Society 2012: 288-291. | Not country of Interest |
| Musa Jaber, M., et al. (2014). "A REVIEW OF ADOPTION OF TELEMEDICINE IN MIDDLE EAST COUNTRIES: TOWARD BUILDING IRAQI TELEMEDICINE FRAMEWORK." Science International 26(5). | Not country of Interest |
| Muzzi, M. (2010). "UNICEF Good Practices in Integrating Birth Registration into Health Systems (2000–2009); Case Studies: Bangladesh, Brazil, the Gambia and Delhi." New York, NY: United Nations Children’s Fund [UNICEF]. | Not country of Interest |
| Myhre, S. L., et al. (2016). "eRegistries: governance for electronic maternal and child health registries." BMC Pregnancy and Childbirth 16(1): 1-11. | Not country of Interest |
| Nassan, M., et al. (2015). "Telepsychiatry for post-traumatic stress disorder: a call for action in the Syrian conflict." The Lancet. Psychiatry 2(10): 866. | Not Design of Interest |
| Nct (2018). "Use of Low-cost mHealth Intervention to Enhance Outcomes of Noncommunicable Diseases Care in Rural and Refugee Settings." Https://clinicaltrials.gov/show/nct03580330. | Not Topic of Interest |
| Nohra, R. G., et al. (2020). "Evaluating the feasibility, acceptability and pre testing the impact of a self-management and tele monitoring program for chronic obstructive pulmonary disease patients in Lebanon: Protocol for a feasibility study." Medicine 99(6): e19021. | Not Design of Interest |
| Oluoch, T., et al. (2012). "The effect of electronic medical record-based clinical decision support on HIV care in resource-constrained settings: a systematic review." International Journal of Medical Informatics 81(10): e83-e92. | Not country of Interest |
| Oomman, N., et al. (2013). "Modernising vital registration systems: why now?" Lancet (London, England) 381(9875): 1336. | Not country of Interest |
| Organization, W. H. (2013). Regional strategy for the improvement of civil registration and vital statistics systems. | Not country of Interest |
| Özdemir, V., et al. (2013). "Crowd-funded micro-grants for genomics and "big data": an actionable idea connecting small (artisan) science, infrastructure science, and citizen philanthropy." Omics: A Journal Of Integrative Biology 17(4): 161-172. | Not Topic of Interest |
| Perakslis, E. D. (2018). "Using digital health to enable ethical health research in conflict and other humanitarian settings." Conflict and health 12(1): 23. | Not country of Interest |
| Phillips, D. E., et al. (2015). "Are well functioning civil registration and vital statistics systems associated with better health outcomes?" The Lancet 386(10001): 1386-1394. | Not country of Interest |
| Rauofi, R., et al. (2014). "Computational Pathology and Telepathology: Poster#069: Telemedicine in north Afghanistan connecting remote area with international medical centres." Pathology 46(Supplement 2): S63. | Not Design of Interest |
| Rossi, L., et al. (2009). "Design and implementation of a hospital information system for the Palestine Red Crescent Society in Lebanon." Eastern Mediterranean Health Journal 15(3): 738-746. | Not Timeframe of Interest |
| Ruzek, J. and C. Yeager (2017). "Internet and mobile technologies: addressing the mental health of trauma survivors in less resourced communities." Global Mental Health 4. | Not country of Interest |
| Ruzek, J. I., et al. (2016). "Mobile mental health interventions following war and disaster." Mhealth 2. | Not country of Interest |
| Shaikh, M. A. (2008). "Nurses' use of global information systems for provision of outreach reproductive health services to internally displaced persons." Prehospital & Disaster Medicine 23(3): s35-38. | Not Timeframe of Interest |
| Sinha, C. and A.-M. Schryer-Roy (2018). "Digital health, gender and health equity: invisible imperatives." Journal of Public Health 40(suppl_2): ii1-ii5. | Not Topic of Interest |
| Sondaal, S. F. V., et al. (2016). "Assessing the effect of mHealth interventions in improving maternal and neonatal care in low-and middle-income countries: a systematic review." PLoS ONE 11(5): e0154664. | Not country of Interest |
| UNICEF and WHO (2018). The Future for Women and Children: UNICEF and WHO joint statement on strengthening civil registration and vital statistics (CRVS) New York and Geneva, United Nations Children’s Fund and World Health Organization. | Not country of Interest |
| Wagner, B., et al. (2012). "Online working alliance predicts treatment outcome for posttraumatic stress symptoms in Arab war-traumatized patients." Depression and anxiety 29(7): 646‐651. | Not country of Interest |
| Walters, T. J. (1996). "Deployment telemedicine: the Walter Reed Army Medical Center experience." Mil Med 161(9): 531-536. | Not country of Interest |
| Waruingi, M., et al. (2009). "Opportunity in delivery of health care over mobile devices in developing countries." AJFAND 9(5): 2. | Not Design of Interest |
| WHO (2010). Telemedicine opportunities and developments in Member States: report on the second global survey on eHealth. . Geneva, World Health Organization. | Not country of Interest |
| WHO (2013). Civil registration and vital statistics 2013: challenges, best practice and design principles for modern systems. Geneva, World Health Organization. | Not country of Interest |
| WHO (2017). Civil registration and vital statistics (CRVS). World Health Organization. | Not country of Interest |
| WHO. Systematic Review of eCRVS and mCRVS Interventions in Low and Middle Income Countries [Internet]. 2013. | Not country of Interest |
| Woodward, A., et al. (2014). "Diffusion of e-health innovations in 'post-conflict' settings: a qualitative study on the personal experiences of health workers." Human Resources for Health [Electronic Resource] 12: 22. | Not country of Interest |
| Wootton, R. (2008). "Telemedicine support for the developing world." Journal of telemedicine and telecare 14(3): 109-114. | Not country of Interest |
| Yugi, J. (2016). "Mr. Yugi: mobile health with large potential in South Sudan." Began with 2015 2: 22. | Not Design of Interest |
| Zatari, D. I. (2002). "Design of a centralized telemedicine model in Palestine." Journal of Telemedicine & Telecare 8 Suppl 2: 96-97. | Not Timeframe of Interest |
