# Supplementary material for: Digital health in fragile states in the Middle East and North Africa (MENA) region: A scoping review of the literature
Source: PLoS One. 2023 Apr 28;18(4):e0285226. doi: 10.1371/journal.pone.0285226 (PMC10146476; doi:10.1371/journal.pone.0285226)
Supplement: S1 Table — (DOCX) [file pone.0285226.s005.docx]

Health providers as intended users **(n=32)**

| **Author Name, Year of Publication, Country of Publication, and Study Design** | **Intended End User** | **Type of Technology Employed** | **Key Findings** |
| --- | --- | --- | --- |
| Abd Ghani, M. K. and M. M. Jaber (2015)^1^.  Iraq Observational Study | Healthcare providers | Telemedicine (willingness to adopt telemedicine) | A framework that includes the influencing factors in adopting telemedicine in Iraq was developed in this study.  **Key influencing** factors in the framework include: (1) privacy; (2) culture; (3) attitude toward telemedicine; (4) benefit; (5) connectivity; (6) IT capability; (7) compatibility; (8) data warehouse; (9) technical support; (10) top management support; (11) cost; (12) policy; (13) upper-level leadership |
| Basma Salameh, R. N., et al. (2019)^2^.  Palestine Observational Study | Healthcare providers | EMR/HIS/Surveillance System (nurses’ readiness for EHR adoption) | **Overall attitude score:** mean score was 59.7 (SD=12.1) -positive attitudes toward EHRs.  -No correlation between age, years of working, and the total means score from the questionnaire (p=130; p=.241, respectively).  -Positive correlation between highest degree obtained and attitude scores (p<.000). |
| Bernasconi, A., et al. (2018)^3^.  Afghanistan  Quasi-experimental Study | Healthcare providers | Digital Algorithms (ALMANACH for child care support) | **-Physical examination appropriateness:** improved from 23.8% [IC% 19.9–28.1] to 84.0% [IC% 77.9–88.6]  **-Treatment appropriateness:** improved from 34.5% [IC% 30.0–39.2] to >85%  **-Antibiotic prescription:** dropped from 86.1% [IC% 82.4–89.2] to less than 30%. |
| Bernasconi, A., et al. (2018)^4^.  Afghanistan Quasi-experimental Study | Healthcare providers | Digital Algorithms  (ALMANACH for child care support) | **-Preventive measures** improved **weighing** increased from 4% pre to 99.6% post, **deworming:** increased from 7.5% to 50.2%, **vitamin A:** increased from 2.7% to 27.5%  **-Antibiotic prescription:** decreased (85.9% vs 38.1%) although diseases in need of antibiotic have increased (34.0% vs 14.9%) |
| Bertani, A., et al. (2012)^5^.  Djibouti  Observational Study | Healthcare providers | Telemedicine (support for pediatric orthopedic surgery) | -**Number of patients referred**: 4% of all pediatric visits were referred to the program (48 consultations for 39 patients, 13 diagnostic advice and 35 for therapeutic advice)  -**Resolving diagnostic uncertainty:** 90% of cases  -**Modification of diseases management:** 37/48 consultations (77%), 49% of which were for surgical indication, 35% for surgical technique, and 16% for both.  -**Agreement:** 2.2/3 between advice given and treatment delivered.  - **Clinical outcomes:** good or very good for 31/38 patients (81%) |
| Bertani, A., et al. (2012)^6^.  Djibouti  Observational Study | Healthcare providers | Telemedicine (support for orthopedic surgery) | - **Number of patients referred**: 157 teleconsultations for 138 patients 67% of which were for neurosurgery and pediatric orthopedics  - **Resolving diagnostic uncertainty:** 78% of cases 29/37.  -**Modification of diseases management:** 69% cases.  - **Clinical outcomes:** good or very good in 86% of the patients |
| Chelala, E., et al. (2015)^7^.  Lebanon  Observational Study | Healthcare providers | Telemedicine (tele-ophthalmology) | -**Number of patients screened**: 119  -**Detection of diabetic retinopathy**: 30.3%  -**Confirmation of diabetic maculopathy:** 7 patients  -The experience revealed that tele-ophthalmology is an effective way for screening diabetic retinopathy in Lebanon. |
| Daniel, F., et al. (2018)^8^.  Lebanon Observational Study | Healthcare providers | Mhealth (physicians’ perception of virtual communication including social media in clinical settings) | **-Any-purpose online app usage pattern:** any application: 100%  Email: 100%, WhatsApp: 96.6%, Facebook: 74.4%, LinkedIn: 38.2% and Twitter: 22.7%; email and LinkedIn used more for professional purposes and other applications for personal purposes.  - **Attitudes and opinions toward the use of online apps in professional settings:** 70.2% believe that it improves communication between physicians, 42.4% believed that it is a beneficial tool for patient education, only 34.0% believed it can be used to improve patient health and treatment compliance, 47.1% believed it can allow patients to share experiences.  -**Barriers**: medicolegal issues and privacy breach based on 78.6% and 71.0% of the respondents respectively  -**Methods of avoiding patient communication:** adjusting privacy settings 60.1%, ignore friend requests 52.1%, ignore email 44.1%, block people 13.4% |
| Doocy, S., et al. (2017)^9^.  Lebanon  Observational Study | Healthcare providers | Mhealth and  EMR/HIS/Surveillance System (treatment guideline + Mhealth tool) | **-Reporting in clinic medical records**: overall low, significant increase in BMI recording, no change in blood pressure readings recorded (p=0.241) or blood sugar tests recording for diabetes patients (p=0.297)  **-Recording on mhealth tool versus clinical medical records:** significant difference in BMI recording: 47.4% versus 15.8%, p<0.001; and blood pressure readings recording: 74.5% versus 40.7%, p<0.001; no significant difference blood glucose test recordings 39.9% versus 34.0%, p=0.185.  **-Patient provider interaction:** improvement in interaction quality and frequency of lifestyle counseling  -**Application use**: 21.7% (CI: 15.9-28.4) of consultations as per patients’ reporting; 48.7% (CI: 32.4-65.2%) received printed output information on medications or lifestyle modification; 23.1% of the patients (CI: 11.1-39.3%) received a summary health record-indicating possible patient underreporting  **-Patient Satisfaction with health workers and clinical interactions:** significant improvement in all questions of the satisfaction questionnaires at mhealth phase compared to baseline and guideline implementation phase (p≤0.01).  **-Provider Perceptions**: mixed based on locations. Advantages: improved patient understanding of lifestyle behaviors and medications, and greater patient satisfaction citing evidence-based treatment algorithms, automation of BMI and risk category calculation, patient printouts, and ability to view the records across facilities as the main benefits. Challenges: time consuming, poor wireless connectivity, technical glitches, and the fact that it was not integrated with existing systems. |
| Doocy, S., et al. (2017)^10^.  Lebanon  Observational Study | Healthcare providers | Mhealth | **Clinical Measurements**  -Compared with baseline reporting of blood pressure (BP) and blood sugar (BS) significantly declined at guideline implementation phase (BP −11.4%, P<.001; BS −6.9%, P=.03) and mhealth phase (−8.4%, P=.001).  -BMI reporting increased at mHealth phase compared to both baseline (8.1%, P<.001) and guidelines phase (7.7%, P<.001).  -Data reported through paper-based versus mhealth: more reporting of BP (74.0% on mhealth vs 38.6% paper records, P<.001), weight (28% mhealth vs 21.8% on paper; P=.10), height (19% mhealth vs 15.8% on paper; P=.25), and BMI (47% mhealth vs 15.8% on paper, P<.001).  **Patient-Provider Interaction**  -Inquiry on medical history: 71.3% enrollment, 87.9% guideline implementation, 99.4% mhealth; Inquiry on medication complications: 36% enrollment, 48.9% guideline implementation P=.04, 95.6% mhealth P<.001; Inquiry on other questions related to illness: 32.0% enrollment, 59.8% guideline implementation P<.001, 98.3% mhealth P<.001; Scheduling a follow-up appointment/specialist referral: 58.0% enrollment, 78.6% guideline implementation P<.001, 97.8% mhealth P<.001  -Counseling about lifestyle behaviors: Smoking cessation counseling was reported for (16.9% patients from the mhealth data versus 11.4% patients from paper records (P=.06)), health dietary habit counseling (77.3% patients from mhealth data vs 8.8% from paper records; P<.001) and physical activity counseling (59.7% patients from app data vs 7.1% from paper records; P<.001). |
| Finlayson, A. E., et al. (2010)^11^. Somalia  Case Study | Healthcare providers | Internet/Websites (clinical bedside teaching between physicians in the UK and students in Somaliland) | -**Infrastructure:** Somali students had sufficient access to the internet at schools and sufficient skills.  -**Website:** stable and reliable  -**North-south teaching:** 95 students in Somaliland learned from 17 tutors in the UK, in weekly teaching sessions.  -**South-north teaching:** 40 global health students in the UK learned about case studies in Somaliland from tutors in Somaliland. 15/16 students reported satisfaction in the tutoring |
| Ghbeis, M. B., et al. (2018)^12^. Syria  Case Study | Healthcare providers | Telemedicine (‘‘Tele-Pediatric Intensive Care’’ program (Tele-PICU)) | **Impact on survival:** Positive impact observed on survival following the introduction of a Tele-PICU.  19 ICU management consultations of which 5 patients survived, 3 patients were transferred, 5 died, and 6 had unknown outcomes. |
| Hashemi, B., et al. (2017)^13^.  Palestine Observational Study | Healthcare providers | Mhealth (Open Data Kit (ODK) mobile application-for children mental illness screening) | **Screening:** 986 children  -The authors also noted that the capability to collect data offline contributed to its success especially in difficult conditions in war-torn areas.  **War experiences reported:** 17 war experiences were identified and participants reported a mean number of 4 experiences (SD 2, range 1–12). Children most frequently reported being injured (69.4%), a family being injured (55.5%), and having their house shelled (53.8%). |
| Helou, S., et al. (2020)^14^.  Lebanon  Observational Study | Healthcare providers | Telemedicine | **-Telehealth Activities before COVID-19**  Physicians used the internet to receive test results from patients (81%) and centers (54%), used phone calls to discuss medical cases with patients (71%), and prescribe medications (46%), provided public awareness using the internet or other media channels (42%) participated in webinars or conferences online (36%) discussed cases in online groups (33%), collaborated with colleagues for research online or over the phone (24%), attended meetings (16%) and trained students (4%) online.  **-Telehealth Activities such as receiving test results, prescribing medications, collaborations and training are more frequently Performed during the Pandemic by physicians**  **-Tools Used for Telehealth before the Pandemic**  Telehealth: WhatsApp (79%), phone calls (77%), and email (76%); Social media: Facebook (26%), YouTube (17%) and Instagram (13%); TV (24%), radio (15%) Video conferencing platforms: Zoom (6%), Skype (5%) Cisco Webex (4%), Microsoft Teams (3%) Slack (0%).  **-Tools More Frequently Used for Telehealth during the Pandemic**  Telehealth: WhatsApp (80%), phone calls (67%), and email (63%); Social media: Facebook (31%), YouTube (16%) and Instagram (12%); TV (13%), radio (12%), Video conferencing platforms: Zoom (74%), Skype (29%) Cisco Webex (31%), Microsoft Teams (27%) Slack (1%).  **-Perceptions Regarding Telehealth pre- versus post-COVID-19**  Increased agreement on the need for telehealth in Lebanon (U= 6671, p < 0.00001); on its role in aligning people’s opinion (U = 8320, p < 0.00001); Increased willingness to invest in this technology (U = 6352, p < 0.00001); Increased agreement that remote consultations are less time-consuming (U = 8360, p = 0.00782), can provide a complete understanding of the patients’ case (U = 8567, p = 0.01778), can be used for first visits (U = 7107, p < 0.00001) are safe for patients (U = 6323, p < 0.00001) and doctors (U = 6805, p < 0.00001), and can provide an equally satisfactory service for patients as face-to-face visits (U = 6345, p < 0.00001). Physicians agreed more on the need to be compensated for their remote consultations (U = 7143, p < 0.00001) and the need to specify a time (U = 6928, p < 0.00001) and place (U = 7452, p = 0.00008) for its provision; No change in perception on regulations (U = 8882, p = 0.05486).  Yet the percentage of the physicians with a perception shift remained a minority (23-41%) |
| Jefee-Bahloul, H., et al. (2016)^15^. Syria  Observational Study | Healthcare providers | Telemedicine (store-and-forward tele-mental health services) | **Providers’ perception**  The majority of the respondents had no experience with tele-mental health, and 50% of the respondents believed that that mental health services can be provided through store and forward tele-mental health services and 77% believed that it would benefit patients. They also identified 3 barriers to the provision of tele-mental health including (1) cultural (68%), (2) financial (84%), and (3) technical barriers (80%). Providers also believed that patients would more willing to be audiotaped then videotaped. |
| Keynejad, R. C. (2016)^16^.  Somalia  Observational Study | Healthcare providers | Telemedicine (e-learning program on psychiatric topics) | **- Knowledge gains:** gains in factual knowledge for Somali students and cross-cultural learning for UK students. Significant positive improvement in ATP scores for Somali students and no change for UK students.  **-Proportion of students considering a career in psychiatry** increased from 55 to 93% for Somali students and no change for UK students.  **-Barriers to Partnership** scheduling times both parties are available in, technical difficulties, other priorities, deadlines and exams, and communication difficulties for planning.  **-Areas of improvement** better logistics management and expansions of topics and specialties.  **-Post-evaluation:** majority had a positive experience and would recommend the program to a friend.  **-Lessons learned:** coordinators should have strong leadership skills, a good working relationship among each other, senior support and swift communication networks. The workload amongst coordinators in both countries should be fairly divided. |
| Keynejad, R., et al. (2013)^17^.  Somalia  Observational Study | Healthcare providers | Telemedicine (e-learning program on psychiatric topics) | Overall the student ratings were good for **enjoyment** 4.31/5, **academic** **helpfulness** 3.56/5, and **interest** **of** **sessions** 4.54/5. |
| Maalim, A. M., et al. (2014)^18^.  Somalia  Observational Study | Healthcare providers | Telemedicine (telemedicine for real-time exchange of information and provision of support between clinicians in Somalia and a physician in Nairobi) | Results showed that using this innovative strategy satisfactory standards of care were met (with adverse outcomes figures at maximum MSF thresholds)  -**Number of admissions** 8584 patients, highest admissions for lower respiratory tract infections (25%), normal deliveries (16%) and diarrheal diseases (715),  Pediatric measles admissions (13% of pediatric admissions) (4 deaths/484 admissions) and adult cases (17% of internal medicine admissions) (2 deaths/315 admissions).  - **Adverse Outcomes** (deaths and absconded): surgical ward (7%), pediatric and internal medicine wards (3%) and maternity (1%). |
| Masrani, A., et al. (2018)^19^. Syria  Case Study | Healthcare providers | Telemedicine (Facebook group and WhatsApp for diagnostic support for Syrian radiologists) | The work was volunteer-based and had no cost, it was sustained for three years and allowed the radiologist to get clinical follow-up on each case (mostly within 24 hours).  Challenges included:  -Unavailability of intravenous contrast for contrast enhanced CT-Scans  -When contrast enhanced CT-Scans were available they were still of low quality due to the absence of protocols, inappropriate timing of intravenous contrast administration and unskilled professionals.  - Poor quality of uploaded pictures  -Lack of archiving systems  -Inability to repeat CT-scans |
| Meri, A., et al. (2019)^20^.  Iraq Modeling Study | Healthcare providers | EMR/HIS/Surveillance System | The effects of system compatibility, system complexity, security, and privacy on physicians’ confirmation and behavioral control were statistically significant. Both confirmation and behavioral control had a positive effect on physicians’ utilization of the technology in the Iraqi hospitals. It is believed that such finding may help to aid the current understanding of cloud health systems in managing health data as well as providing the necessary recommendations for policy makers to direct healthcare professionals to continuously consider the use of modern information and communications technology in the workplace. |
| Mohamed, K. G., et al. (2014)^21^.  Sudan Observational Study | Healthcare providers | Telemedicine  (2 year Master’s program for family medicine-including telemedicine (peer-to-peer support) EMR, and e-learning) | In relation to digital health, the program leveraged information and communication technology including telemedicine (WebEx, virtual classrooms) for peer-to-peer decision support and e-learning and an electronic filing system to facilitate training and service. |
| Mohamed, K. G., et al. (2015)^22^.  Sudan Observational Study | Healthcare providers | Telemedicine and EMR/HIS/Surveillance System (ICT implementation and utilization in a 2 year Master’s for family medicine) | **-Number of telemedicine consultations:** 3808 consultations, 74% conducted by females, mainly for consultations related to internal medicine (32.6%) pediatrics (21.8%).  **-Trainees’ Evaluation of Telemedicine Use**: The majority of trainees agreed on the importance of telemedicine for their patients (100%), that it is a good training tool (100%), and that their patients are satisfied with it (89%). 81% stated that they took teleconsultations in the presence of their patients while 20% were concerned about losing patients’ confidence.  -**New patients’ EMR creation:** 165993 by 125 doctors  -**Trainees’ Evaluation of EMRs Use:** 97% agreed that EMRs are important for patient care, 72% agreed that they registered most/all their patients on EMR, while 74% agreed that patients are unhappy when doctors use EMR during consultations.  **-E-Learning:** 240 lectures and 29 meetings, for family medicine (38%), internal medicine (23%), pediatrics (15%), obstetrics and gynecology (8%), and surgery (8%).  -**Trainees’ Evaluation of E-Learning Use** 91% of the users agreed that online lectures are good teaching methods 71.4% agreed on the ease of combining work with the training activities. |
| Shadi, S., et al. (2016)^23^.  Lebanon Observational Study | Healthcare providers | EHealth (primary care providers’ Ehealth readiness) | -**Access to computers and comfort using them:** Most healthcare professionals had access to computers (94%) and were comfortable using them (90%).  -**Appropriateness:** those who were more comfortable using computers were more likely to report the appropriateness of e-health and management support for it (p = 0.002 & p = 0.009, respectively).  -**Management support:** other healthcare providers were more likely to report management support for e-health than physicians (p =0.007). Full-timers were also more likely to report support than part-timers (p = 0.014).  -**Efficacy:** those who were very comfortable using computers were more likely to report efficacy than those who comfortable (p = 0.001 and p = 0.01, respectively), and not comfortable (p =0.000 & p = 0.008, respectively)  -Those who had access to computers at the centers were more likely to believe they had the skills to implement e-health (p = 0.01).  -Other healthcare providers were more likely to report efficacy than nurses (p = 0.005) and were more likely to believe they had the skills to implement e-health (p = 0.005).  - **Personally beneficial:** other healthcare providers and physicians were more likely to report that e-health were personally beneficial than nurses (p = 0.005 and p = 0.001, respectively). Those who had access to computers were also more likely to report that e-health is personally beneficial (p = 0.022)  -General result demonstrates readiness of healthcare providers to adopt e-health |
| Saleh, S., et al. (2018)^24^.  Lebanon  Observational Study | Healthcare providers | Mhealth (netbook for NCD screening) | The study demonstrated the effectiveness of an e-health tool (netbook) in facilitating the screening process of non-communicable diseases namely hypertension and diabetes, the scheduling of appointments, and referrals in outreach activities for underserved communities. |
| Tirmizi, S. N., et al. (2017)^25^.  Afghanistan Quasi-experimental Study | Healthcare providers | Mhealth (blended learning approach for mental health) | - **Overall Knowledge:** a significant improvement in scores from 45% pre-intervention to 63% post-intervention.  - **Background knowledge:** 30% pre to 40% post (P value =0.001)  - **Knowledge of symptoms**: 25% pre to 44% post (P value =0.001)  -**Knowledge related to causes of depression** from 22% to 51% (P value =0.008) (4) treatment knowledge of depression from 29% to 35% (P value =0.01).  - **Gains in scores**: significant difference between intervention and control districts (16.06 versus 6.80; P =0.009).  - **Difference in knowledge** between intervention and control groups was significant for the background and causes of depression categories. |
| Zachariah, R., et al. (2012)^26^.  Somalia Observational Study | Healthcare providers | Telemedicine  (between clinical staff in Somalia and a specialist pediatrician in Kenya for information exchange) | - **Number of referrals**: 346 of 3920 admissions  - **Change in case management**: 64% of the referred cases.  - **Detection of a life-threatening condition after being initially missed:** in 25% of the referred cases.  -**Local clinicians’ capacity in complicated disease management**: significant decrease in alternations to initial case management for meningitis and convulsions (92–29%, X² for trend 10.1, P = 0.001), lower respiratory tract infection (75–47%, X² for trend 5.1, P = 0.02) and complicated malnutrition (86–40%, X² from trend, 9.4, P = 0.002).  -**Adverse outcomes:** 7.6% in 2010 (Pre) to 5.4% in 2011 (post) (odds ratio 0.70, 95% CI: 0.57–0.88, P ≤0.001).  - **Perceived usefulness**: (7/7) perceived useful, improving risk signs identification (7 ⁄7); management protocols and prescription practices (6 ⁄7); the relationship of solidarity with specialist colleagues (5 ⁄7). |
| Elhadi, M., et al. (2021).^27^  Libya  Observational Study | Healthcare providers | Telemedicine (telehealth services in Libya) | *377 (56%), 582 (86.5%), and 566 (82.6%) physicians had high awareness, knowledge, and attitude levels for telemedicine, respectively; only 248 (36.8%) participants had adequate or high computer skills.* |
| Ibrahem, et al. (2021).^28^  Sudan  Case study | Healthcare providers | Telemedicine (Social media/e-learning) | The COVID‐19 ACIM program was successfully delivered and demonstrated that delivering high quality virtual medical education to a low‐resource country is feasible.  The use of freely available social media tools can help deliver medical education to a low‐resource country. |
| Karah, N., et al. (2022)^29^.  Syria  Case study | Healthcare providers | Telemedicine/ELR | TCM module, along with the TmHC platform, offers a tool to the few laboratory staff remaining on the ground in Syria to share diagnostic results with remote international experts, facilitating the provision of laboratory-guided infection treatments. |
| Nsouli, R., & Vlachopoulos, D. (2021) ^30^.  Lebanon  Observational study | Healthcare providers | Information and Communication Technology (ICT) | Stress, lack of experience, lack of knowledge, limited skills, and poor infrastructure, are factors that prevent educators from using ICT in their teaching practices.  Nursing faculty members’ attitudes can be categorized into three groups: faculty with positive attitudes who are pioneers in the use of ICT, staff with neutral attitudes—who tend to be followers by nature—and faculty members with negative attitudes—resisters who oppose ICT use. |
| Stauch, G., et al. (2022).^31^  Afghanistan  Case study | Healthcare providers | Telemedicine | Telemedicine is possible and necessary even in countries with a high potential for conflict; it is integrated into routine care by the local doctors. |
| Zaidi, S., Kazi, et al. (2020).^32^  Afghanistan  Observational Study | Healthcare providers | Mhealth (The Hayat app) | The Hayat app being in the local language, supplemented with a simple interface, made it easy for the CHWs to adopt and use it. It saved time, organized the work routine, removed the need to work with manual data registers, and promoted accountability. |

1. Abd Ghani MK, Jaber MM. Willingness to Adopt Telemedicine in Major Iraqi Hospitals: A Pilot Study. *Int J Telemed Appl*. 2015;2015:136591. doi:<https://dx.doi.org/10.1155/2015/136591>

2. Basma Salameh PhD RN, Linda L. Eddy PhD RA, Ahmad Batran PhD RN, Asma Hijaz RN, Shorook Jaser RN. Nurses' Attitudes Toward the Use of an Electronic Health Information System in a Developing Country. article. *SAGE Open Nursing*. 04/01/ 2019;5doi:10.1177/2377960819843711

3. Bernasconi A, Crabbe F, Rossi R, et al. The ALMANACH Project: Preliminary results and potentiality from Afghanistan. *International Journal of Medical Informatics*. Jun 2018;114:130-135. doi:<https://dx.doi.org/10.1016/j.ijmedinf.2017.12.021>

4. Bernasconi A, Crabbé F, Raab M, Rossi R. Can the use of digital algorithms improve quality care? An example from Afghanistan. Article. *PLoS ONE*. 2018;13(11):1-12. doi:10.1371/journal.pone.0207233

5. Bertani A, Menguy P, Delmas JM, et al. [Assessment of surgical teleconsultations in a developing country: two years of experience in Djibouti]. *Medecine Et Sante Tropicales*. 2012;22(4):405-408. doi:10.1684/mst.2012.0112

6. Bertani A, Launay F, Candoni P, Mathieu L, Rongieras F, Chauvin F. Teleconsultation in paediatric orthopaedics in Djibouti: evaluation of response performance. *Orthop Traumatol Surg Res*. Nov 2012;98(7):803-7. doi:<https://dx.doi.org/10.1016/j.otsr.2012.03.022>

7. Chelala E, Saleh N, Dirani A, et al. [Screening of diabetic retinopathy and maculopathy in Lebanese population using retinography and SD-OCT: The role of telemedicine]. *J Med Liban*. Apr-Jun 2015;63(2):59-65. DEPISTAGE de la RETINOPATHE et de la MACULOPATHIE DIABETIQUE au LIBAN par RETINOGRAPHIE et par SD-OCT: INTERET de la TELEMEDECINE.

8. Daniel F, Jabak S, Sasso R, Chamoun Y, Tamim H. Patient-Physician Communication in the Era of Mobile Phones and Social Media Apps: Cross-Sectional Observational Study on Lebanese Physicians' Perceptions and Attitudes. *JMIR medical informatics*. Apr 6 2018;6(2):e18. doi:10.2196/medinform.8895

9. Doocy S, Paik K, Lyles E, et al. Pilot Testing and Implementation of a mHealth tool for Non-communicable Diseases in a Humanitarian Setting. *PLoS Curr*. Jun 05 2017;9:05. doi:<https://dx.doi.org/10.1371/currents.dis.e98c648aac93797b1996a37de099be74>

10. Doocy S, Paik KE, Lyles E, et al. Guidelines and mHealth to Improve Quality of Hypertension and Type 2 Diabetes Care for Vulnerable Populations in Lebanon: Longitudinal Cohort Study. *JMIR MHealth and UHealth*. Oct 18 2017;5(10):e158. doi:<https://dx.doi.org/10.2196/mhealth.7745>

11. Finlayson AE, Baraco A, Cronin N, et al. An international, case-based, distance-learning collaboration between the UK and Somaliland using a real-time clinical education website. Research Support, Non-U.S. Gov't. *J Telemed Telecare*. 2010;16(4):181-4. doi:<https://dx.doi.org/10.1258/jtt.2010.004004>

12. Ghbeis MB, Steffen KM, Braunlin EA, et al. Tele-Pediatric Intensive Care for Critically Ill Children in Syria. *Telemedicine Journal And E-Health: The Official Journal Of The American Telemedicine Association*. 2018;24(8):621-623. doi:10.1089/tmj.2017.0216

13. Hashemi B, Ali S, Awaad R, Soudi L, Housel L, Sosebee SJ. Facilitating mental health screening of war-torn populations using mobile applications. *Soc Psychiatry Psychiatr Epidemiol*. 01 2017;52(1):27-33. doi:<https://dx.doi.org/10.1007/s00127-016-1303-7>

14. Helou S, El Helou E, Abou-Khalil V, et al. The Effect of the COVID-19 Pandemic on Physicians' Use and Perception of Telehealth: The Case of Lebanon. Research Support, Non-U.S. Gov't. *International Journal of Environmental Research & Public Health [Electronic Resource]*. 2020;17(13):06.

15. Jefee-Bahloul H, Duchen D, Barkil-Oteo A. Attitudes Towards Implementation of Store-and-Forward Telemental Health in Humanitarian Settings: Survey of Syrian Healthcare Providers. *Telemed J E Health*. Jan 2016;22(1):31-5. doi:<https://dx.doi.org/10.1089/tmj.2015.0021>

16. Keynejad RC. Global health partnership for student peer-to-peer psychiatry e-learning: Lessons learned. Review

Research Support, Non-U.S. Gov't. *Global health*. 12 03 2016;12(1):82.

17. Keynejad R, Ali FR, Finlayson AET, et al. Telemedicine for peer-to-peer psychiatry learning between U.K. and Somaliland medical students. *Academic Psychiatry: The Journal Of The American Association Of Directors Of Psychiatric Residency Training And The Association For Academic Psychiatry*. 2013;37(3):182-186. doi:10.1176/appi.ap.11080148

18. Maalim AM, Zachariah R, Khogali M, et al. Supporting 'medicine at a distance' for delivery of hospital services in war-torn Somalia: how well are we doing? Evaluation Studies

Research Support, Non-U.S. Gov't. *Int Health*. Mar 2014;6(1):70-3. doi:<https://dx.doi.org/10.1093/inthealth/iht035>

19. Masrani A, Mamoun I, Tarabishy B, Tarabishy A, Arabi M. Delivering Humanitarian Teleradiology Services to Besieged Areas in Syria. *J*. Aug 2018;15(8):1175-1177. doi:<https://dx.doi.org/10.1016/j.jacr.2018.03.052>

20. Meri A, Hasan MK, Danaee M, et al. Modelling the utilization of cloud health information systems in the Iraqi public healthcare sector. Article. *Telematics and Informatics*. 03/01/March 2019 2019;36:132-146. doi:10.1016/j.tele.2018.12.001

21. Mohamed KG, Hunskaar S, Abdelrahman SH, Malik EM. Scaling up family medicine training in Gezira, Sudan - a 2-year in-service master programme using modern information and communication technology: a survey study. *Human resources for health*. Jan 21 2014;12:3. doi:10.1186/1478-4491-12-3

22. Mohamed KG, Hunskaar S, Abdelrahman SH, Malik EM. Telemedicine and E-Learning in a Primary Care Setting in Sudan: The Experience of the Gezira Family Medicine Project. *int*. 2015;2015:716426. doi:<https://dx.doi.org/10.1155/2015/716426>

23. Shadi S, Rawya K, Mohamad A, Maysa B. Readiness of healthcare providers for eHealth: the case from primary healthcare centers in Lebanon. article. *BMC Health Services Research, Vol 16, Iss 1, Pp 1-11 (2016)*. 2016;(1):1. doi:10.1186/s12913-016-1896-2

24. Saleh S, Alameddine M, Farah A, et al. eHealth as a facilitator of equitable access to primary healthcare: the case of caring for non-communicable diseases in rural and refugee settings in Lebanon. *Int J Public Health*. Jun 2018;63(5):577-588. doi:<https://dx.doi.org/10.1007/s00038-018-1092-8>

25. Tirmizi SN, Khoja S, Patten S, et al. Mobile-based blended learning for capacity building of health providers in rural Afghanistan. *mHealth*. 2017;3:14. doi:10.21037/mhealth.2017.04.01

26. Zachariah R, Bienvenue B, Ayada L, et al. Practicing medicine without borders: tele-consultations and tele-mentoring for improving paediatric care in a conflict setting in Somalia? Research Support, Non-U.S. Gov't. *Trop Med Int Health*. Sep 2012;17(9):1156-62. doi:<https://dx.doi.org/10.1111/j.1365-3156.2012.03047.x>

27. Elhadi M, Elhadi A, Bouhuwaish A, et al. Telemedicine Awareness, Knowledge, Attitude, and Skills of Health Care Workers in a Low-Resource Country During the COVID-19 Pandemic: Cross-sectional Study. *Journal of Medical Internet Research*. 2021;23(2):e20812.

28. Ibrahem A, Elzein H, Adlan A. Social media facilitates COVID‐19 training in a low‐resource country. Article. *Medical Education*. 2021;55(11):1303-1304. doi:10.1111/medu.14653

29. Karah N, Antypas K, Al-toutanji A, et al. Teleclinical Microbiology: An Innovative Approach to Providing Web-Enabled Diagnostic Laboratory Services in Syria. Journal article. *American Journal of Clinical Pathology*. 2022;157(4):554-560. doi:10.1093/ajcp/aqab160

30. Nsouli R, Vlachopoulos D. Attitudes of nursing faculty members toward technology and e-learning in Lebanon. *BMC Nurs*. 2021;20:116.

31. Stauch G, Raoufi R, Sediqi A, et al. [Experiences with telepathology in northern Afghanistan : A 10-year success story]. Review. *Pathologie (Heidelberg, Germany)*. 2022;43(4):303-310. Erfahrungen mit Telepathologie in Nordafghanistan : Eine 10-jahrige Erfolgsgeschichte.

32. Zaidi S, Kazi AM, Riaz A, et al. Operability, Usefulness, and Task-Technology Fit of an mHealth App for Delivering Primary Health Care Services by Community Health Workers in Underserved Areas of Pakistan and Afghanistan: Qualitative Study. Research Support, Non-U.S. Gov't. *Journal of Medical Internet Research*. 2020;22(9):e18414.
