# Supplementary material for: Digital health in fragile states in the Middle East and North Africa (MENA) region: A scoping review of the literature
Source: PLoS One. 2023 Apr 28;18(4):e0285226. doi: 10.1371/journal.pone.0285226 (PMC10146476; doi:10.1371/journal.pone.0285226)
Supplement: S2 Table — (DOCX) [file pone.0285226.s006.docx]

**Clients n=25**

| **Author Name, Year of Publication, Country of Publication, and Study Design** | **Intended End User** | **Type of Technology Employed** | **Key Findings** |
| --- | --- | --- | --- |
| Alhaidari, T., et al. (2018)^2^.  Iraq Experimental Study | Clients | Mhealth (SMS program for antenatal care) | -**Number of antenatal care visits**: the median number of antenatal care visits was significantly higher in intervention group compared to control (median in intervention group =4 versus 2 in control group, P < 0.001).  -**Patient Satisfaction**: subjects most frequently voted favorably (>85%) for the following items: “The client recommends this program for other pregnant women”, “Personal rating for the message as a whole” and “Obtained benefit from the messages” and had less frequent favorable responses (<70%) on “Receiving the message in the proper time of the day” and “Messages encouraged the client to visit the PHCC at the scheduled time”.  -**Patient/provider interaction:** 60.8% of the participants made at least one phone call to inquire about pregnancy problems, content of an SMS message, labor and puerperium. |
| Ali Saare, M., et al. (2019)^3^. Iraq Literature Review | Clients | Mhealth (factors affecting mhealth use among older adults | -**Identified factors**: “perceived usefulness, perceived ease of use, subjective norm, and facilitating conditions.”  -**Predictors of mobile health adoption** perceived usefulness, perceived ease of use, and subjective norm.  -**Predictors of intention to use**: perceived usefulness, subjective norm, and facilitating condition.  - Perceived ease of use studies were strongly correlated with intention to use mobile health applications on older adults  (double check this please) |
| Ali, A. O. A. and M. H. Prins (2019)^4^.  Sudan Quasi-experimental Study | Clients | Mhealth (mhealth for tuberculosis treatment adherence-SMS) | - **Default rate:** Patients in the intervention group had a lower default rate than control group yet the difference between the groups was not statistically significant (default rate = 6.8% in intervention versus 10.8% in control group; P= 0.563; OR: 1.673, 95% C.I. 0.521-5.374).  - **Cure rate:** Patients in the intervention group had higher cure rate than control group (78.4% in intervention versus 59.5 in control; P= 0.020; OR: 2,472, 95% CI: 1.133 – 5.434).  - **Knowledge of tuberculosis and its treatment** was the same at the beginning of the experiment between the two groups (OR ranging between 0.78 and 1.32). At the end of the experiment knowledge in the intervention group better than the control group, but was only significantly better 3/5 knowledge characteristics. |
| Bardus, M., et al. (2019)^5^.  Lebanon Observational Study | Clients | Mhealth (applications for weight management) | **-Overall quality**: moderate for the six applications: highly functional but not engaging.  -**Subjective quality scores** low indicating that the users’ intentions to reuse was low.  -As engagement and subjective scores were positively related it was suggested that application developers develop applications that are more engaging while holding constant other aspects of quality such as functionality, aesthetics, and information quality. |
| Ben-Zeev, D., et al. (2017)^6^.  Palestine Observational Study | Clients | Mhealth (assessment of the viability of mhealth approaches as an alternative to traditional mental health services in rural areas) | - **Mobile phone ownership**: the majority owned mobile phones, the majority of which were smartphones.  -**Internet and Wi-Fi access**: majority had access  - Approximately all mobile phone owners used social media.  - **Prevalence of mental health problems**: over half of the region was estimated to have mental health problems  -**Willingness to use mHealth tools for mental health support**: majority expressed willingness to use mhealth including: bi-directional text messaging, mobile applications; unidirectional support texts, or web-intervention. |
| Binyam, B., et al. (2020)^7^.  Palestine Observational Study | Clients | Mhealth (Targeted client communication (TCC) using text messages for maternal and child health) | -A Targeted client communication (TCC) using text messages was co-designed with users based on behavior change theories and concepts following in-depth interviews with women and healthcare providers.  -Knowledge and awareness gaps were identified and tailored text messages were developed to address these gaps and increase the utilization of antenatal care.  -The intensity of these messages were modified based on the mother’s identified health risks in the electronic health records. |
| Istepanian, R. S., et al. (2014)^8^. Iraq Quasi-experimental Study | Clients | Mhealth (feasibility study for Mhealth tool for the management of diabetes-glucose monitor connected via Bluetooth to a mobile application) | The authors’ concluded that adopting a Mhealth tool for the management of diabetes is feasible in this challenging context as the administration of this tool lowered the HBA1C of the intervention group. |
| Lakkis, N. A., et al. (2011)^9^.  Lebanon  Experimental study | Clients | Mhealth (brief SMS message versus longer informative message for routine mammography uptake) | -**Response rate:** 31.2% did the mammography test after receiving the message  -**Brief vs longer message efficacy:** results indicated that both messages were equally effective (30.7% and 31.6% of group receiving the brief message and that receiving the longer respectively) (Chi-square test, p-value≥0.05) |
| Lebrun, V., et al. (2020)^10^.  Afghanistan Quasi-experimental Study | Clients | Mhealth (Mobile Alliance for Maternal Action program- voice message + SMS text) | -SMS messages were less likely to be missed than voice messages especially by female participants.  -The vast majority of the participants reported benefit especially in increased knowledge.  - Including both parents was reported as beneficial by the vast majority of participants. Joint decision making among the 2 parents regarding their child health increased.  -Correct knowledge significantly increased for the majority of participants of all but one maternal and child health measure at follow up. |
| McCarthy, O. L., et al. (2019)^11^.  Palestine Experimental study | Clients | Mhealth (Text-message intervention for contraception) | -**%** **of participants that reported that at least one method of contraception is acceptable** greater for the intervention group (2.34 times odds)  -**Odds of contraception use:** greater odds of participants having an IUD, injection, implant, patch or long-acting reversible contraception method was observed in intervention group  - **Knowledge and attitude**: intervention group exhibited higher mean knowledge, showed more likeliness to agree that their friends should use contraception, and more willingness to use contraception in the future. |
| Melissa Joanne Harper, S., et al. (2020)^12^.  Lebanon Quasi-experimental Study | Clients | Mhealth (pilot test of e-mental health intervention based on the WHO Step-by-Step intervention) | -Improved depression symptoms.  -A significant difference in the Wilcoxon signed ranks test  -Positive client satisfaction  -Patient satisfaction with relationship with non-specialist support person (e-helper)  -E-helpers noted the need for more training on complex cases  -Interviewee suggested that material should be also tailored to younger and single individuals, and that they should be easier to use.  -Analysis of website data showed that those who dropped-out did so before the start of the intervention. |
| Saleh, S., et al. (2018)^13^.  Lebanon Experimental study | Clients | Mhealth (educational text-messages) | -**Health Outcomes**: Improvement in blood pressure control, HbA1c levels, and decrease in mean systolic blood pressure.  -**Separate Regression analysis controlling for age, gender, and setting showed:**  -Intervention group had lower odds of poor HbA1c levels, and increased odds of controlled blood pressure post-intervention (I am not sure of this).  -Females were at lower odds of poor HbA1c control  -Age was associated in the control group with a decrease in HbA1c  - The HbA1c of patients in rural areas was more controlled than those in camps  (Please read full results section and validate this I am not sure I captured everything). |
| Saleh, S., et al. (2018)^14^.  Lebanon Observational Study | Clients | Mhealth (SMS based intervention for NCD lifestyle modifications) | -**Usefulness**: the vast majority of respondents agreed that the SMSs were useful and easy to read and understand.  -**Change in behavior:** The majority of respondents reported change of behavior related to the receipt of SMSs- change was statistically significant across settings (rural areas versus camps)  -Those who received SMSs and did not read them were more likely to be women, aged 76 or older, unemployed, illiterate or only could read and write. |
| Talhouk, R., et al. (2016)^15^.  Lebanon Observational Study | Clients | EHealth/Digital Health in general (informing the design of digital technologies for antenatal care in refugee setting) | The study identified key considerations that should be taken into account while designing the technology including:  -Refugees’ health believes and experiences  -Their literacy level (to consider for instance whether to send text messages or audio recording)  -Refugees’ perceptions during previous encounters with providers  -Their hierarchal and familial structures (such as the role of the settlement “*sheisha”*). |
| Wagner, B., et al. (2012)^16^.  Iraq Experimental study | Clients | Internet/Websites (internet based intervention for post-traumatic stress disorder (PTSD)) | - **Total scores for Posttraumatic Diagnostic Scales (PDS)** dropped significantly from pre to post treatment, 29.13 to 13.86 (t(14)=−6.72, p<0.0001, d=1.57), where intrusions dropped from 8.80 to 3.27 (t(14)=5.29, p<0.0001, d=1.44), avoidance dropped from 11.13 to 5.80 (t(14)=−4.60, p<0.0001, d=1.23), and hyperarousal, from 9.20 to 4.80 (t(14)=5.83, p<0.0001, d=1.43).  -**Symptoms on the HSCL-25 depression and anxiety** subscales were reduced from 2.78 to 1.85 for depression (t(14)=4.99, p<0.0001, d=1.51) and from 2.93 to 2.02 for anxiety (t(14)=4.49, p<0.0001, d=1.50).  -**The quality of life scale (EUROHIS) increased** from 19.07 to 26.20 (t(14)=−5.18, p<0.0001, d=1.17).  -**Percentage of patients with clinically significant complaints** dropped from 86% Pretreatment to 33% posttreatment for Posttraumatic Diagnostic Scales (PDS), from 93% to 46% for depression (HSCL-25), and from 93% to 60% for anxiety (HSCL-25). |
| Yamin, F., et al. (2018)^17^.  Afghanistan Observational Study | Clients | Mhealth (perception of afghan women in utilizing mhealth for maternal and child care) | -**Mobile phone ownership**: the majority of the participants owned mobile phones (59.2%-142/240) and 220 participants used mobile phones routinely (91.7%).  -**Familiarity with phone services:** 70.0% were familiar with both Voice calls and SMS while 13.2% were only familiar with SMS.  -**Agreement that mhealth can be used to support health:** the majority of participants agreed that mobile phones can be used for supporting health with the majority agreeing that phones can be used for appointments (80.3%) and counseling (79.7%).  -**Openness to receiving SMS:** The majority (87.1%) were open to receiving messages about nutrition, pregnancy, breastfeeding, vaccination, hygiene and others.  -**Preferred communication method:** Their preferred method was through automated voice calls (80.8%) sent weekly (55.0%)  -**Willingness to call a helpline**: the majority were willing to call a free helpline for support (80.4%), were open to receiving reminders about national immunization days (72.1%), children’s vaccinations (89.6), and tetanus vaccinations (93.3%).  -**Openness to receiving reminders**: the majority were open to receiving reminders about perinatal care visits (93.8%), 2 days before (47.7%), and preferred voice calls (80.0%) over SMS (2.5%). |
| Bogale, B., et al. (2021).^18^  Palestine  Experimental Study | Clients | Mhealth (TCC via SMS for ANC services) | The TCC intervention via SMS did not increase pregnancy-related worries among recipients. There was no difference in women’s satisfaction with the ANC services between intervention and control arms. |
| Cuijpers, P., et al. (2022). ^19^  Lebanon  Experimental Study | Clients | Mhealth (WHO-guided digital mental health intervention, Step-by-Step) | The guided, digital intervention was effective in reducing depression in displaced people in Lebanon. The guided WHO Step-by-Step intervention we examined should be made available to communities of displaced people that have digital access. |
| Cuijpers, P., et al. (2022).^20^  Lebanon  Experimental Study | Clients | Mhealth (WHO-guided digital mental health intervention, Step-by-Step) | Guided digital mental health interventions can be effective in the treatment of depression in communities exposed to adversities in LMICs |
| Elhadi, M., et al. (2021)^21^  Libya  Observational Study | Clients | Telemedicine (assess the usability of telehealth services in Libya and to provide an overview of the current COVID-19 scenario. | This study revealed high levels of usability and willingness to use the telemedicine system as an alternative modality to in-person consultations among the Libyan residents in this study. |
| Heim, E., et al. (2021)^22^  Lebanon  Experimental Study | clients | Mhealth (internet-based guided self-help intervention for adults with depression called Step-by-Step | The research design, methods and procedures are feasible and acceptable in the context of Lebanon and can be applied in the RCTs. Preliminary findings suggest that Step-by-Step may be effective in reducing symptoms of depression and anxiety and improving functioning and well-being. |
| Ibrahim Abu-El-Noor, N., et al. (2021)^23^  Palestine  Experimental Study | clients | Mhealth (mobile phone app to evaluate the level of adherence to treatment regimens among hypertensive patients in the Gaza Strip) | - **Adherence to treatment**: Patients in the intervention group showed a significantly better adherence in total score (change in intervention= -7.42 versus -2.72 in control P=0.000), medication adherence (change in intervention= -3.91 versus -1.92 in control P=0.000) and diet adherence (change in intervention= -2.63 versus -1.25 in control P=0.000).  - **Appointment adherence scores**: no significant difference was observed among intervention and control group (P=0.052) |
| Lorenzetti, L., et al. (2022).^24^  Afghanistan  retrospective descriptive | clients | Mhealth (Mobile for Reproductive Health (m4RH) | The number of CMs listened to across all menus increased with time. The basic m4RH family planning menu was most popular, with callers most frequently selecting information on intrauterine contraceptive devices. Nine types of promotional approaches were implemented. Compared against call volume, SMS blast promotion was the most productive promotional approach, radio broadcasts had modest increases, and social media and interpersonal communication demonstrated no clear change. |
| Pia, A. Z., et al. (2021).^25^  Lebanon  Observational Study | clients | Telehealth (Embrace Lifeline for suicidal individuals) | The helpline is effectively reducing distress and suicidal ideation, across a wide sample of callers. |
| Nohra, R. G., et al. (2022).^26^  Lebanon  Quasi-experimental | clients | Telehealth/Telemedicine  (pretest the effectiveness of an educational and telemonitoring program in a sample COPD patients) | All 15 participants who agreed to participate in this intervention found the program adequate and acceptable for addressing COPD-related issues. Regarding adoption, all participants declared having no difficulty explaining to others the content of the education sessions and that they would suggest this program to other COPD patients. In terms of effectiveness, six patients had improving in quality of life scores, and five patients had lower anxiety and depression scores compared to baseline measures. A knowledge assessment was done at the end of each session, showing an increase in knowledge for all participants. |

1. Abu-El-Noor NI, Aljeesh YI, Bottcher B, Abu-El-Noor MK. Impact of a mobile phone app on adherence to treatment regimens among hypertensive patients: A randomised clinical trial study. *European journal of cardiovascular nursing : journal of the Working Group on Cardiovascular Nursing of the European Society of Cardiology*. 2020:1474515120938235. doi:10.1177/1474515120938235

2. Alhaidari T, Amso N, Jawad TM, et al. Feasibility and acceptability of text messaging to support antenatal healthcare in Iraqi pregnant women: a pilot study. *J Perinat Med*. Jan 26 2018;46(1):67-74. doi:<https://dx.doi.org/10.1515/jpm-2016-0127>

3. Ali Saare M, Hussain A, Seng Yue W. Conceptualizing Mobile Health Application Use Intention and Adoption Among Iraqian Older Adults: From the Perspective of Expanded Technology Acceptance Model. article in journal/newspaper. 2019;doi:10.3991/ijim.v13i10.11285

4. Ali AOA, Prins MH. Mobile health to improve adherence to tuberculosis treatment in Khartoum state, Sudan. *Journal of Public Health in Africa*. 2019;10(2):1101.

5. Bardus M, Ali A, Demachkieh F, Hamadeh G. Assessing the Quality of Mobile Phone Apps for Weight Management: User-Centered Study With Employees From a Lebanese University. Research Support, Non-U.S. Gov't. *JMIR MHealth and UHealth*. 2019;7(1):e9836.

6. Ben-Zeev D, Fathy C, Jonathan G, et al. mHealth for mental health in the Middle East: Need, technology use, and readiness among Palestinians in the West Bank. *Asian J Psychiatr*. Jun 2017;27:1-4. doi:<https://dx.doi.org/10.1016/j.ajp.2017.02.010>

7. Binyam B, Kjersti M, Brian OD, et al. Development of a targeted client communication intervention to women using an electronic maternal and child health registry: a qualitative study. article. *BMC Medical Informatics and Decision Making*. 01/01/ 2020;20(1):1-12. doi:10.1186/s12911-019-1002-x

8. Istepanian RS, Mousa A, Haddad N, et al. The potential of m-health systems for diabetes management in post conflict regions a case study from Iraq. Research Support, Non-U.S. Gov't. *Conf Proc IEEE Eng Med Biol Soc*. 2014;2014:3650-3. doi:<https://dx.doi.org/10.1109/EMBC.2014.6944414>

9. Lakkis NA, Atfeh AM, El-Zein YR, Mahmassani DM, Hamadeh GN. The effect of two types of sms-texts on the uptake of screening mammogram: a randomized controlled trial. Comparative Study; Journal Article; Randomized Controlled Trial. *Preventive medicine*. 2011;53(4‐5):325‐327. doi:10.1016/j.ypmed.2011.08.013

10. Lebrun V, Dulli L, Alami SO, et al. Feasibility and Acceptability of an Adapted Mobile Phone Message Program and Changes in Maternal and Newborn Health Knowledge in Four Provinces of Afghanistan: Single-Group Pre-Post Assessment Study. *JMIR MHealth and UHealth*. 2020;8(7):e17535.

11. McCarthy OL, Zghayyer H, Stavridis A, et al. A randomized controlled trial of an intervention delivered by mobile phone text message to increase the acceptability of effective contraception among young women in Palestine. *Trials*. Apr 23 2019;20(1):228. doi:10.1186/s13063-019-3297-4

12. Melissa Joanne Harper S, Jinane Abi R, Pim C, et al. Step-by-Step, an E-Mental Health Intervention for Depression: A Mixed Methods Pilot Study From Lebanon. article. *Frontiers in Psychiatry*. 02/01/ 2020;10doi:10.3389/fpsyt.2019.00986

13. Saleh S, Farah A, Dimassi H, et al. Using Mobile Health to Enhance Outcomes of Noncommunicable Diseases Care in Rural Settings and Refugee Camps: Randomized Controlled Trial. *JMIR MHealth and UHealth*. Jul 13 2018;6(7):e137. doi:<https://dx.doi.org/10.2196/mhealth.8146>

14. Saleh S, Farah A, El Arnaout N, et al. mHealth use for non-communicable diseases care in primary health: patients' perspective from rural settings and refugee camps. *J Public Health (Oxf)*. Dec 01 2018;40(suppl_2):ii52-ii63. doi:<https://dx.doi.org/10.1093/pubmed/fdy172>

15. Talhouk R, Mesmar S, Thieme A, et al. Syrian refugees and digital health in Lebanon: Opportunities for improving antenatal health. 2016:331-342.

16. Wagner B, Schulz W, Knaevelsrud C. Efficacy of an Internet-based intervention for posttraumatic stress disorder in Iraq: a pilot study. Research Support, Non-U.S. Gov't. *Psychiatry Res*. Jan 30 2012;195(1-2):85-8. doi:<https://dx.doi.org/10.1016/j.psychres.2011.07.026>

17. Yamin F, Kaewkungwal J, Singhasivanon P, Lawpoolsri S. Women's Perceptions of Using Mobile Phones for Maternal and Child Health Support in Afghanistan: Cross-Sectional Survey. *JMIR MHealth and UHealth*. Apr 10 2018;6(4):e76. doi:<https://dx.doi.org/10.2196/mhealth.9504>

18. Bogale B, Mørkrid K, Abbas E, et al. The effect of a digital targeted client communication intervention on pregnant women's worries and satisfaction with antenatal care in Palestine-A cluster randomized controlled trial. Journal article. *PloS one*. 2021;16(4):e0249713. doi:10.1371/journal.pone.0249713

19. Cuijpers P, Heim E, Abi Ramia J, et al. Effects of a WHO-guided digital health intervention for depression in Syrian refugees in Lebanon: a randomized controlled trial. Journal Article; Clinical Trial Protocol. *PLoS medicine*. 2022;19(6):e1004025. doi:10.1371/journal.pmed.1004025

20. Cuijpers P, Heim E, Ramia JA, et al. Guided digital health intervention for depression in Lebanon: randomised trial. *Evidence Based Mental Health*. 2022;16:16.

21. Elhadi M, Msherghi A, Elhadi A, et al. Utilization of Telehealth Services in Libya in Response to the COVID-19 Pandemic: Cross-sectional Analysis. *JMIR Medical Informatics*. 2021;9(2):e23335.

22. Heim E, Ramia JA, Hana RA, et al. Step-by-step: feasibility randomised controlled trial of a mobile-based intervention for depression among populations affected by adversity in Lebanon. Journal article. *Internet interventions*. 2021;24doi:10.1016/j.invent.2021.100380

23. Ibrahim Abu-El-Noor N, Ibrahim Aljeesh Y, Bottcher B, Khalil Abu-El-Noor M. Impact of amobile phone app on adherence to treatment regimens among hypertensive patients: A randomised clinical trial study. Article. *European Journal of Cardiovascular Nursing*. 2021;20(5):428-435. doi:10.1177/1474515120938235

24. Lorenzetti L, Plourde KF, Rastagar SH, et al. Analyzing program data and promotional approaches to inform best practices from a mobile phone-based reproductive health message program in Afghanistan. *Digital Health*. 2022;8:20552076221089801.

25. Pia AZ, Farah EY, Lila ZK, et al. Evaluation of Lebanon's National Helpline for Emotional Support and Suicide Prevention: Reduction of Emotional Distress among Callers. Article. *Intervention (15718883)*. 2021;19(2):1-11.

26. Nohra RG, Chaaban T, Sacre H, et al. Evaluating the Feasibility and Pretesting the Impact of an Educational and Telemonitoring Program for COPD Patients in Lebanon. *International Journal of Copd*. 2022;17:949-965.
