# Supplementary material for: Digital health in fragile states in the Middle East and North Africa (MENA) region: A scoping review of the literature
Source: PLoS One. 2023 Apr 28;18(4):e0285226. doi: 10.1371/journal.pone.0285226 (PMC10146476; doi:10.1371/journal.pone.0285226)
Supplement: S3 Table — (DOCX) [file pone.0285226.s007.docx]

Data services (**n=15**)

| **Author Name, Year of Publication, Country of Publication, and Study Design** | **Intended End User** | **Type of Technology Employed** | **Key Findings** |
| --- | --- | --- | --- |
| Ahmed, R., et  al. (2018)[1]  Sudan  Observational  Study | Data services | Mhealth (Questionnaire data collected using smartphones versus paper-based) | -**Agreement between paper based and smartphone based questionnaire:** strong to slight. For the 204 variables (Kappa 1.00-0.02 and inter-rater coefficient 1.00 -0.12).  -**Errors**: smartphones collected questionnaires resulted in fewer errors (82.5% of errors occurred on paper based, 10.5% on smartphone-based questionnaires, and7% occurred in both formats (X² (3, n = 100) = 64, p < .001)). |
| Anam, L. S., et al. (2019)[2]. Yemen Observational Study | Data services | EMR/HIS/Surveillance System (Comparison between two systems for malaria surveillance) | -**System Performance**:  System 1: overall poor (42%); scored averagely for flexibility (71%), poorly for simplicity (48%), acceptability (34%), representativeness (52%), and stability (46%).  System 2: overall performance score was good (87%), data quality (100%), acceptability (97%), and flexibility (100%) were excellent; simplicity was good (85%); representativeness (72%) and stability (67%) were average. |
| Askar, A., et al. (2018)[3].  Somalia Case Study | Data services | EMR/HIS/Surveillance System (Assessment of the Somalian health information system) | **-Areas of weakness** policy, strategic planning, information fragmentation, poor infrastructure, under-skilled labor, unstainable funding, absence of civil registration, no integration of private sector data, incapacity for data analysis, poor data dissemination, and poor data utilization.  -**Scoring for resources** 43% (i.e. present but inadequate).  - **Scoring for indicators** 72%  - **Scoring of data sources** 21% (i.e. not adequate): health and disease records scored at 60%, census at 10%, vital statistics at 5%, and population based sources at 9%.  -**Scoring of data management** 52% (i.e. adequate)  -**Scoring of** **information management** scored at 22% (i.e. not adequate).  - **Scoring of data dissemination and use** 50% (i.e. present but not adequate) |
| Asmar, M. K., et al. (2016)[4].  Lebanon  Case Study | Data services | EMR/HIS/Surveillance System (lessons learned from statistics reports preparation) | **Lessons Learned:**  -The importance of stakeholder partnership  -The need for an adequate management system, human resources, and systems and procedures.  -The need for a portal for information exchange among stakeholders and the public.  -The importance of clear guidelines, definitions, and standardized forms for the data collection and analysis process |
| El-Nour, A. E. A. M., et al. (2016)[5].  Sudan  Observational Study | Data services | EMR/HIS/Surveillance System (identification of needs to strengthen state-level health information systems) | **Identified needs:** challenges in infrastructure, human skills and coordination. |
| Emma, D., et al. (2017)[6].  Syria  Case Study | Data services | EMR/HIS/Surveillance System (lessons from operational experience and literature review on HIS) | **Lessons Learned:**  -Health information systems were established de novo to respond to humanitarian needs (independent from government systems).  -Social media facilitated data collection in remote areas in Syria |
| Haskew, J., et al. (2015)[7].  Sudan  Case Study | Data services | Mhealth (implementation of mobile information technology) | -**Usefulness** **in national round of polio immunization program**: useful in the planning, implementation, and evaluation; particularly in mapping, supervision and surveying. |
| Kamadjeu, R. and C. Gathenji (2017)[8]. Somalia Case Study | Data services | Internet/Websites (description of the design and implementation of an electronic dashboard- Somalia Polio Room) | -Centers located in multiple locations exchanged data with the central coordination unit in real-time to provide the data.  -The dashboard displayed key outbreak response performance indicators to inform decision making. |
| Venkateswaran, M., et al. (2018)[9].  Palestine Observational Study | Data services | EMR/HIS/Surveillance System (paper-based health information system versus individual-level data eRegistry) | Paper-based system resulted in an **underestimation in key maternal conditions:**  -Fundal height (20% versus 0.01%)  -Rh-negative blood group (6.8% versus 1.4%)  -Anemia with hemoglobin<9.5 g/dl (6% versus 0.6%)  -Mal-presentation at term (1.3% versus 0.03%). |
| Ashworth, H., et al. (2022)[10].  Lebanon  Observational | Data services | EMR (Electronic health records (EHRs) | We report the successes and challenges from 12 months of Hikma Health EHR deployment in a mobile clinic providing care to Syrian refugees in Bekaa Valley, Lebanon. Successes include the EHR’s ability to (1) increase clinical efficacy by providing detailed patient records, (2) be adaptable to the threats of COVID-19, and (3) improve organizational planning. Lessons learned include technical fixes to methods of identifying patients through name or their medical record ID. Conclusions: As the number of displaced people continues to rise globally, it is imperative that solutions are created to help maximize the health care they receive. Free, open-sourced, and adaptable EHRs can enable organizations to better provide for displaced populations. |
| Mergenthaler, C., et al. (2021)[11]  Afghanistan  Observational Study | Data services | Computer-aided personal interviewing (CAPI) | The added value of CAPI over PAPI was primarily in time efficiencies gained during data collection and processing, although only conducted in relatively secure areas. CAPI also resulted in improved acceptability while preserving data quality, despite higher costs. Overall our data provide evidence to support the scale up of digital survey technology in future surveys in Afghanistan and other fragile and conflict affected settings. |
| Mobaied, S. (2020)[12].  Syria  Case study | Data services | GIS (The Risk of Vulnerability to Covid-19 in War Zones Index “Id_Covid19_WZ index”) | GIS can identify and map vulnerable areas in any conflict zone in the world. The resulting map can be used to manage the pandemic in this region by preparing the most vulnerable zones with the necessary health facilities and protective measures. |
| Shadeed, S., & Alawna, S. (2021)[13].  Palestine  Case study | Data services | GIS (Geographic Information system for COVID-19) | **GIS show areas vulnerable to COVID-19** (e.g. very high vulnerability Nablus, Jerusalem, and Hebron governorates; Tulkarm, Ramallah & Al-Bireh and Jenin governorates are highly vulnerable; 82 % of the West Bank population are under high to very high COVID-19 vulnerability classes; 14% and 4 % are medium and low to very low vulnerable, respectively) |
| Youssef, D., et al. (2022) [14].  Lebanon  Case study | Data services | HIS (district health information system (DHIS-2) | Implementation of DHIS-2 improved timeliness and completeness for aggregated data reporting. |
| Pan African Medical, J. (2022)[15]  Soudan  Case study | Data services | EMR/HIS/Surveillance System  (real-time monitoring tool for polio vaccination) | The study had shown that real-time information has significantly improved the smooth conduct of the immunization campaign processes through identifying gaps, and challenges in the field and can be utilized in similar resource settings including complex and humanitarian. It has demonstrated the capability of mobile phones using ODK for data collection and linked to a Power BI dashboard for enhanced supervision and transparency. |

1. Ahmed, R., et al., *A comparison of smartphone and paper data-collection tools in the Burden of Obstructive Lung Disease (BOLD) study in Gezira state, Sudan.* PLoS ONE [Electronic Resource], 2018. **13**(3): p. e0193917.

2. Anam, L.S., et al., *Evaluation of Two Malaria Surveillance Systems in Yemen Using Updated CDC Guidelines: Lessons Learned and Future Perspectives.* Inquiry, 2019. **56**: p. 46958019880736.

3. Askar, A., M. Ardakani, and R. Majdzade, *Bridging gaps in health information systems: a case study from Somaliland, Somalia.* Eastern Mediterranean Health Journal, 2018. **23**(11): p. 764-773.

4. Asmar, M.K., J.S. Yeretzian, and A. Rady, *Compiling comprehensive national health statistics in a fragmented health information system: lessons learned from Lebanon.* Eastern Mediterranean Health Journal, 2016. **22**(1): p. 52-57.

5. El-Nour, A.E.A.M., M.K. Elnimeiri, and A.M.O. Abbas, *Towards a Digitized and Integrated Health Information System in Sudan: Assessment of Readiness at State Level.* Sudan Journal of Medical Sciences, 2016. **11**(2): p. 55-59.

6. Emma, D., et al., *The role of public health information in assistance to populations living in opposition and contested areas of Syria, 2012–2014.* Conflict and Health, Vol 11, Iss 1, Pp 1-12 (2017), 2017(1): p. 1.

7. Haskew, J., et al., *Use of Mobile Information Technology during Planning, Implementation and Evaluation of a Polio Campaign in South Sudan.* PLoS ONE [Electronic Resource], 2015. **10**(8): p. e0135362.

8. Kamadjeu, R. and C. Gathenji, *Designing and implementing an electronic dashboard for disease outbreaks response - Case study of the 2013-2014 Somalia Polio outbreak response dashboard.* The Pan African medical journal, 2017. **27**(Suppl 3): p. 22.

9. Venkateswaran, M., et al., *Comparing individual-level clinical data from antenatal records with routine health information systems indicators for antenatal care in the West Bank: A cross-sectional study.* Plos One, 2018. **13**(11): p. e0207813-e0207813.

10. Ashworth, H., et al., *A Free, Open-Source, Offline Digital Health System for Refugee Care.* JMIR Medical Informatics, 2022. **10**(2): p. e33848.

11. Mergenthaler, C., et al., *Going digital: added value of electronic data collection in 2018 Afghanistan Health Survey.* Emerging Themes in Epidemiology, 2021. **18**(1): p. 16.

12. Mobaied, S., *A new method for identifying and mapping areas vulnerable to Covid-19 in an armed conflict zone: Case study north-west Syria.* MethodsX, 2020. **7**: p. 101091.

13. Shadeed, S. and S. Alawna, *GIS-based COVID-19 vulnerability mapping in the West Bank, Palestine.* International Journal of Disaster Risk Reduction, 2021. **64**.

14. Youssef, D., et al., *Converting the existing disease surveillance from a paper-based to an electronic-based system using district health information system (DHIS-2) for real-time information: the Lebanese experience.* BMC Health Services Research, 2022. **22**(1): p. 395.

15. *Erratum: Real-time monitoring of a circulating vaccine-derived poliovirus outbreak immunization campaign using digital health technologies in South Sudan doi: 10.11604/pamj.2021.40.200.31525.* Pan Afr Med J, 2022. **41**: p. 105.
