# Supplementary material for: Digital health in fragile states in the Middle East and North Africa (MENA) region: A scoping review of the literature
Source: PLoS One. 2023 Apr 28;18(4):e0285226. doi: 10.1371/journal.pone.0285226 (PMC10146476; doi:10.1371/journal.pone.0285226)
Supplement: S4 Table — (DOCX) [file pone.0285226.s008.docx]

Health providers and clients as intended users (**n=11**)

| **Author Name, Year of Publication, Country of Publication, and Study Design** | **Intended End User** | **Type of Technology Employed** | **Key Findings** |
| --- | --- | --- | --- |
| Abdi, Y. A. and J. Y. Elmi (2011)^1^.  Somalia Case Study  (Commentary) | Clients Healthcare providers | Telemedicine  (Skype-based tele-psychiatry for the provision of mental health services) | - **Number of consultations:** 132 patients were consulted with.  -**Diagnosis:** Males were most frequently diagnosed with schizophrenia followed by Khat related psychosis, females were most frequently diagnosed with mood disorders, and children were mostly diagnosed with epilepsy. |
| Abi Ramia, J., et al. (2018)^2^. Lebanon Observational Study | Clients Healthcare providers | Internet/Websites  (“Step-by-Step” internet based mental health intervention) | **-Edits for contextualization** shortening of the content, adding a video representation to the content, and increasing the focus on mood lifting activities.  The mode of contact with the patients was also contextualized to the local norms and activities were updated based on suggestions from community members. |
| Antoun, J., et al. (2019)^3^.  Lebanon Observational Study | Clients Healthcare providers | EMR/HIS/Surveillance System  (electronic medical records and patient-physician communication) | -**Quality of communication**: Patients reported that it did not affect the quality of communication with their healthcare provider.  **Themes emerged from interviews:**  -The necessity of electronic medical records use  -The efficiency of electronic medical records in record keeping and information retrieval  -The ability of physicians to balance between computer use and communicating with patients  -Patients were not disturbed by physician computer use while communicating with them. |
| Gladys, N. H.-A., et al. (2020)^4^.  Lebanon  Observational Study | Clients Healthcare providers | EMR/HIS/Surveillance System (electronic patient portals) | -**Intention to use:** 52% of the patients (with a 3.2-3.7 mean intention to use per feature) and 73% of providers (82.4% to post educational material, 70.6 to encourage their patients to record weight and other measures on it, and 76.5% intended to advice patients to administrative tasks such as schedule appointments).  -**Perceived usefulness:** 88% of the patients and 82% of providers  -**Perceived ease of use:** 42% of the patients (in all aspects of posting information, communicating with the physician, and finding information) and 77% of providers (significant difference). Perceived ease of use increased with education (.37 SD per 1 SD increase educational level) and decreased with age (0.4 SD per 1SD increase in age).  -**Social influence:** 95% of the patients and 86% of provider with a significant difference in privacy concerns patients (M= 2.13, SD = .9), p < .001, CI [−2.16, −.91] versus (M= 3.67, SD = 1.11) for providers  -Perceived ease of use increased perceive usefulness, and perceived usefulness increased intention to use more than perceived ease of use. |
| Khoja, S., et al. (2015)^5^.  Afghanistan Case Study | Clients Healthcare providers | Mhealth and Telemedicine  (Educational SMS + blended learning + Ehealth screening and treatment for mental health) | **-Number of Messages sent:** 10,000 text messages (to 1200 adolescents).  **-E-learning**: 33 blended learning sessions for health providers-attended by 8,006 community members and 596 health providers.  **-Number of Mental Health Mobile Application Users** 95 community health workers and 25 facility based health workers (serving ˃100,000 people).  **-Screening and Diagnosis through mobile application guidelines:** 1954 screened and 160 mental health cases diagnosed. |
| Khoja, S., et al. (2016)^6^.  Afghanistan Quasi-experimental | Clients Healthcare providers | Mhealth and Telemedicine (Educational SMS + blended learning + Ehealth screening and treatment for mental health) | **-Improvement in awareness about mental health in the community:** Agreement that mental health conditions are treatable: Intervention (I) 94% vs 86% Control (C) (odds ratio (OR) =2.52; p=0.0027); Responding correctly regarding symptoms of depression: I 90% vs 73% C (OR=2.17; p<0.0001); Understood that use of drugs and alcohol can be a sign of depression: I 88% vs 78% C (OR=2.03; p=0.0027).; Denying myths on psychosis: I 68% vs 27% C (OR=5.66; p=0.0001); Responding correctly regarding signs of psychosis: I 64% vs 55% C (OR=1.56; p<0.0001); Understood that past traumatic events can be a cause of PTSD: I 77% vs 53% C (OR=2.99; p=0.0001); Understood the importance of early I can be favorable for PTSD prognosis: I 85% vs 58% C (OR=4.14; p=0.0001); Considering awareness regarding PTSD important: I 83% vs 58% C (OR=3.41; p=0.0001); Disagreeing that mental health patients are largely to blame for their own condition: I 93% vs 85% C (OR=2.33; p=0.003); Believing that people should be kind and caring with mental health patients: I 92% vs 80% C (OR=2.76; p=0.0001).  **-Reduction in stigma in the community:** Agreeing that patients should not be discouraged from sharing their problems: I 87% vs 52% C (OR=6.22; p<0.0001); Believing that mental health patients should not be attacked or threatened: I 87% vs 48% C (OR=6.82; p< 0.0001); Believing in equal rights and respect for healthy people and mental health patients: I 60% vs 48% C (OR=6.22; p< 0.0001); Avoiding or discriminating against mental health patients: I 8% vs 75% C (OR=29.8; p< 0.0001); Insulting mental health patients: I 7% vs 61% C (OR=22.94; p< 0.0001).  **-SMS technology and improvement in awareness among young adults:** finding messages easy to understand 96% at mid-term evaluation (MTE) vs 66% at baseline (B) (chi2=71.9; p=0.001), requesting SMS continuation 97% at MTE vs 66% at B (chi2=78; p<0.001), understanding mental health issues 99% at MTE vs 65% at B.  **-Mental health guidelines and improvement in referrals:** information relevant and up-to-date 82% at MTE vs 53% at B (chi2=14.3; p=0.001), and 77% of providers reported that guidelines changed their practice. |
| Salem, R., et al. (2020)^7^.  Lebanon Observational Study | Clients and  Healthcare providers | Telemedicine a (distance support program (telemedicine) for home palliative care) | -**Caregivers’** **perception** appreciated the support namely for information and guidance, emotional support, and access to providers although some still stated preference for home visits.  -**Providers’ perception** stated that the support program was efficient, but stated that it is safer and easier to support after the patient have received at least one home visit, that for providers to be successful they should have extensive experience in palliative care, that the ability of the caregiver to communicate clearly and continuously is important, and finally that expectations need to be clarified at the beginning of the session (including the limit of services that can be provided through telemedicine). |
| Elahssan, R., et al. (2022)^8^.  Sudan  Narrative review | Clients Healthcare providers | Telemedicine (telemedicine and virtual clinics) | Despite some understandable limitations, virtual clinics can represent a feasible alternative to conventional outpatients’ clinics in this challenging time of COVID-19 pandemic. Virtual clinics when used appropriately can allow continuation of patient’s care, improve accessibility and reduces the risk of nosocomial transmission. In Sudan’s setting, there is huge potential for TH although this would be limited by administrative support and cultural acceptability. |
| Itani, R., et al. (2021)^9^  Lebanon  Observational study | Clients Healthcare providers | Telehealth/Telemedicine  (pharmaceutical care provided by community pharmacists to suspected high-risk COVID-19 patients using telehealth) | An unsatisfactory level of preparedness through means of telehealth technology was evident. This resulted in the quality of pharmaceutical-care services provided to high-risk patients via telehealth to be below expectations. |
| Naal, H., et al. (2021)^10^.  Lebanon  Narrative review | Clients Healthcare providers | Telemedicine (Telemental Health) | TMH implementation has its own barriers, including lack of research and training on TMH and financial, technological, and infrastructural limitations. However, the usefulness, adaptability and scalability of TMH, the expressed interest in telehealth by the Ministry of Public Health and the growing global momentum around TMH are opportunities for adoption in Lebanon. Partnerships between CBOs, private, and governmental institutions are essential to develop, implement, and sustain TMH programs. |
| Relyea, B., et al. (2021)^11^.  Iraq  Observational study | Clients Healthcare providers | Mhealth (mobile phone interventions for obstetric care) | several challenges contributed to the second delay in accessing obstetric care in northern Iraq, where conflict and displacement have burdened the health system. A mobile phone-based app to organize and coordinate referrals was deemed acceptable by the stakeholders in this setting and has potential to reduce delays relating to transportation. |

1. Abdi YA, Elmi JY. Internet based telepsychiatry: a pilot case in Somaliland. *Med Confl Surviv*. Jul-Sep 2011;27(3):145-50.

2. Abi Ramia J, Harper Shehadeh M, Kheir W, et al. Community cognitive interviewing to inform local adaptations of an e-mental health intervention in Lebanon. *Global Mental Health (Cambridge, England)*. 2018;5:e39-e39. doi:10.1017/gmh.2018.29

3. Antoun J, Hamadeh G, Romani M. Effect of computer use on physician-patient communication using interviews: A patient perspective. *International Journal of Medical Informatics*. 2019;125:91-95.

4. Gladys NH-A, Jumana A, Karim B, Sani H, Houry N. Users’ acceptance of electronic patient portals in Lebanon. article. *BMC Medical Informatics and Decision Making*. 02/01/ 2020;20(1):1-12. doi:10.1186/s12911-020-1047-x

5. Khoja S, Khan MA, Husyin N, et al. Improving Mental Health Care for Young Adults in Badakshan Province of Afghanistan Using eHealth. *Stud Health Technol Inform*. 2015;209:46-50.

6. Khoja S, Scott R, Husyin N, et al. Impact of simple conventional and Telehealth solutions on improving mental health in Afghanistan. *J Telemed Telecare*. Dec 2016;22(8):495-498.

7. Salem R, El Zakhem A, Gharamti A, Tfayli A, Osman H. Palliative Care via Telemedicine: A Qualitative Study of Caregiver and Provider Perceptions. *Journal of Palliative Medicine*. 2020;23:23.

8. Elahssan R, Shariff F, M OG, Yousif TI. Telehealth application in Sudan: requirements and potential benefits. Review. *Sudanese Journal of Paediatrics*. 2022;22(1):5-9.

9. Itani R, Khojah HMJ, Jaffal F, Rahme D, Karout L, Karout S. Provision of pharmaceutical care to suspected high-risk COVID-19 patients through telehealth: a nationwide simulated patient study. *BMC Health Services Research*. 2021;21(1):997.

10. Naal H, Mahmoud H, Whaibeh E. The potential of telemental health in improving access to mental health services in Lebanon: Analysis of barriers, opportunities, and recommendations. Article. *International Journal of Mental Health*. 2021;50(3):218-233. doi:10.1080/00207411.2020.1863743

11. Relyea B, Wringe A, Afaneh O, et al. Stakeholders' Perspectives on the Challenges of Emergency Obstetric Referrals and the Feasibility and Acceptability of an mHealth Intervention in Northern Iraq. *Frontiers in Global Womens Health*. 2021;2:662256.
