# Supplementary material for: Digital health in fragile states in the Middle East and North Africa (MENA) region: A scoping review of the literature
Source: PLoS One. 2023 Apr 28;18(4):e0285226. doi: 10.1371/journal.pone.0285226 (PMC10146476; doi:10.1371/journal.pone.0285226)
Supplement: S5 Table — (DOCX) [file pone.0285226.s009.docx]

Health system or organization managers as intended users **(n=10)**

| **Author Name, Year of Publication, Country of Publication, and Study Design** | **Intended End User** | **Type of Technology Employed** | **Key Findings** |
| --- | --- | --- | --- |
| Al-Fadhli, A. A., et al. (2017)^1^.  Yemen Observational Study | Health system or resource managers | Telemedicine (Assessment of Yemeni health system needs) | -**Yemeni health system needs** include a lack and an inequitable distribution of facilities, funding, health professional and resources.  -**Rural areas needs** included three categories needs services (lack of facilities and bad transportation), human resources (lack of continuing education and high rate of medical errors), and health management information (no health information system).  -The analysis of the findings showed that telehealth might be an effective tool in meeting rural area needs (for instance for elearning). |
| Durrani, H., et al. (2012)^2^.  Afghanistan Observational Study | Health system or resource managers | EHealth/Digital Health in general (health needs and Ehealth readiness) | **-Health needs** of French Medical Institute for Children (FMIC) and  Bamyan Provincial Hospital (BPH): provision of care needs; capacity building needs; and information management (e.g. paper based information management).  **-EHealth readiness** reported by healthcare providers (HCP) was the same at both hospitals while higher in FMIC than BPH as per managers (M) reports  Core readiness: FMIC (3.9/5 as per HCP and 4.6/5 as per M) versus BPH (4.1 HCP and 2.8 M); 4.2 FMIC versus 3.2 HCP (only available for managers); learning readiness 3.6 for both hospitals (per HCP only); societal readiness (3.8 as per HCP and 4.0 as per M) versus BPH (3.2 HCP and 2.0 M); policy readiness (4.0 as per HCP and 4.2 as per M) versus BPH (3.HCP and 2.0 M) |
| Leonardo Emberti, G., et al. (2020)^3^.  Iraq Case Study | Health system or resource managers | EMR/HIS/Surveillance System (Health Monitoring System) | -**System Implementation and Use:** 59 primary health centers and public hospitals were using the system,  **-Training**: 258 health operators were trained on using it, and 734 doctors, statisticians, and health managers were trained on public health.  -The authors noted the importance of establishing multiple stakeholder partnership for the success of the project implementation. |
| Talhouk, R., et al. (2020)^4^.  Lebanon  Observational Study | Health system or resource managers | EHealth/Digital Health in general (integrating health technology in primary healthcare centers) | **Challenges identified:**  - The unavailability of time and technological resources in some centers.  - The interviewees’ perception of refugee technological illiteracy (varied among interviewees)  - The hesitation to invest in technology for this population as it is viewed as a mobile population |
| Yugi, J. and H. M. Buesseler (2016)^5^. Sudan  Case Study | Health system or resource managers | Mhealth (cheap SMS application for weekly surveillance reporting) | - **User Perception:** interesting and easy to use.  -**Timeliness of data reporting:** increased  **Challenges:**  -Not having mobile network coverage in some centers.  -The unavailability of dedicated staff in some centers. |
| Halwani, J., & Mouawad, D. (2021)^6^.  Lebanon  Observational study | Health system or resource managers | EMR/HIS/Surveillance System | The hospitals surveyed had partly implemented and continuously tried to apply some e-health technologies, but there were no real medical records for patients. Various challenges were faced for full e-health technology implementation in Lebanon: primarily cost, followed by some personnel resistance, lack of legislation and common standards, and the necessity for continual training. |
| Hassan, I. A., et al. (2022)^7^.  Iraq  Case study | Health system or resource managers | EMR/HIS/Surveillance System | The study found successful utilization of the EHS in the outpatient's department of the hospital and underutilization in the emergency department, and other departments due to many barriers especially the not well-trained staff in the hospital and the workload from a high number of patients and problems in the system itself and its update. |
| Salman, K. J., et al. (2021)^8^.  Iraq  Case study | Health system or resource managers | GIS (Geographic Information system) | there is a decline in the level of health services in terms of geographical location and the preparation of medical and technical staff and management and the type of services provided by these establishments to the citizens when compared with local and global standards. |
| Venkateswaran, M., et al. (2022)^9^  Palestine  Experimental | Health system or resource managers | HIS/EMR | Clinical decision support for antenatal care in the eRegistry was superior for most process outcomes but had no effect on the adverse health outcomes. |
| Venkateswaran, M., et al. (2022)^10^.  Palestine  Experimental | Health system or resource managers | HIS/EMR | Markedly less time (plausibly a saving of at least 18%) was spent on health information management in eRegistry clinics compared to those that use paper-based documentation. |

1. Al-Fadhli AA, Othman M, Al-Jamrh BA. Towards Improving the Healthcare Services in Least Developed Countries: A Case of Health Needs Assessment for Telehealth in Yemen. Springer; 2017:605-615.

2. Durrani H, Khoja S, Naseem A, Scott RE, Gul A, Jan R. Health needs and eHealth readiness assessment of health care organizations in Kabul and Bamyan, Afghanistan. Multicenter Study

Research Support, Non-U.S. Gov't. *East Mediterr Health J*. Jun 2012;18(6):663-70.

3. Leonardo Emberti G, Faiq BB, Stefania M, et al. Supporting Iraqi Kurdistan Health Authorities in Post-conflict Recovery: The Development of a Health Monitoring System. article. *Frontiers in Public Health*. 01/01/ 2020;8doi:10.3389/fpubh.2020.00007

4. Talhouk R, Akik C, Araujo-Soares V, et al. Integrating Health Technologies in Health Services for Syrian Refugees in Lebanon: Qualitative Study. *Journal of Medical Internet Research*. 2020;22(7):e14283.

5. Yugi J, Buesseler HM. Text messaging app improves disease surveillance in rural South Sudan. *Mhealth*. 2016;2:8. doi:<https://dx.doi.org/10.21037/mhealth.2016.03.01>

6. Halwani J, Mouawad D. Implementation of e-health innovative technologies in North Lebanon hospitals. *Eastern Mediterranean Health Journal*. 2021;27(9):892-898.

7. Hassan IA, Al-Azzawi AAM, Talal ML. Evaluation of e-Health records system: Case study albatool teaching hospital in Iraq. Article. *AIP Conference Proceedings*. 2022;2386(1):1-6. doi:10.1063/5.0066977

8. Salman KJ, Salah MA, Al-Hussain YA. Adopting geographical infornation systems (GIS) for investigation the reality of health care centers in Samawa city, Iraq. Article. *AIP Conference Proceedings*. 2021;2404(1):1-7. doi:10.1063/5.0069685

9. Venkateswaran M, Ghanem B, Abbas E, et al. A digital health registry with clinical decision support for improving quality of antenatal care in Palestine (eRegQual): a pragmatic, cluster-randomised, controlled, superiority trial. Comparative Study

Randomized Controlled Trial

Research Support, Non-U.S. Gov't. *The Lancet Digital Health*. 2022;4(2):e126-e136.

10. Venkateswaran M, Nazzal Z, Ghanem B, et al. eRegTime-Time Spent on Health Information Management in Primary Health Care Clinics Using a Digital Health Registry Versus Paper-Based Documentation: Cluster-Randomized Controlled Trial. *JMIR Formative Research*. 2022;6(5):e34021.
